# Inhibition of lethal inflammatory responses through the targeting of membrane-associated Toll-like receptor 4 signaling complexes with a Smad6-derived peptide

**DOI:** 10.15252/emmm.201404653

**Published:** 2015-03-12

**Authors:** Youn Sook Lee, Jin Seok Park, Su Myung Jung, Sang-Doo Kim, Jun Hwan Kim, Jae Young Lee, Kyeong Cheon Jung, Mizuko Mamura, Sangho Lee, Seong-Jin Kim, Yoe-Sik Bae, Seok Hee Park

**Affiliations:** 1 Department of Biological Sciences, Sungkyunkwan UniversitySuwon, Korea; 2 Department of Pathology, College of Medicine, Seoul National UniversitySeoul, Korea; 3 Department of Molecular Pathology, Tokyo Medical UniversityTokyo, Japan; 4 Department of Internal Medicine, Kyungpook National University School of MedicineDaegu, Korea; 5 CHA Cancer Institute, CHA UniversitySeoul, Korea; 6 Samsung Advanced Institute for Health Sciences and Technology, Sungkyunkwan UniversitySeoul, Korea

**Keywords:** inflammation, Pellino-1, sepsis, Smad6, TLR4

## Abstract

We have previously reported that Smad6, one of the inhibitory Smads of transforming growth factor-β (TGF-β)/bone morphogenetic protein (BMP) signaling, inhibits Toll-like receptor (TLR) 4 signaling by disrupting the Pellino-1-mediated TLR4 signaling complex. Here, we developed Smaducin-6, a novel membrane-tethered palmitic acid-conjugated Smad6-derived peptide composed of amino acids 422–441 of Smad6. Smaducin-6 interacted with Pellino-1, located in the inner membrane, thereby disrupting the formation of IRAK1-, RIP1-, IKKε-mediated TLR4 signaling complexes. Systemic administration of Smaducin-6 showed a significant therapeutic effect on mouse TLR4-mediated inflammatory disease models, cecal-ligation–puncture (CLP)-induced sepsis, and lipopolysaccharide-induced endotoxemia, by inhibiting pro-inflammatory cytokine production and apoptosis while enhancing neutrophil migration and bacterial clearance. Our findings provide clues to develop new peptide-based drugs to target Pellino-1 protein in TLR4 signaling pathway for the treatment of sepsis.

## Introduction

Smad6 has been originally identified as one of the inhibitory Smads to inhibit transforming growth factor (TGF)-β/bone morphogenic protein (BMP) signaling through reduced phosphorylation of Smad2 and Smad5, competition with Smad4, and downregulation of Smad4 with Smurf1 (Imamura *et al*, 1997; Hata *et al*, 1998; Morén *et al*, 2005). Beyond its conventional role as a negative feedback regulator of TGF-β/BMP signaling, Smad6 negatively regulates Toll-like receptor (TLR) 4 signal through dual mechanisms: disruption of the Pellino-1-mediated TLR4 signaling complex (Choi *et al*, 2006) and selective degradation of MyD88 (Lee *et al*, 2011).

Pellino-1, encoded by the *Peli1* gene, is an adaptor protein originally identified in *Drosophila* that binds to *Pelle*, the *Drosophila* homolog of mammalian interleukin-1 receptor-associated kinase (IRAK) (Grosshans *et al*, 1999). There are three members of the mammalian Pellino family, Pellino-1, Pellino-2, and Pellino-3 (Moynagh, 2014). Early experiments in cell lines showed that Pellino-1 is capable of interacting with IRAK1 and IRAK4 and thus an involvement in interleukin-1 receptor (IL-1R) signaling and TLR signaling as a scaffolding protein was suggested (Jiang *et al*, 2003; Choi *et al*, 2006; Schauvliege *et al*, 2006; Moynagh, 2009, 2014). However, Pellino-1-deficient mice revealed that Pellino-1 is involved in the TLR4 and TLR3 signaling pathways through its E3 ubiquitin ligase activity, but dispensable for IL-1R signaling (Chang *et al*, 2009; Moynagh, 2014). Furthermore, recent studies indicate that Pellino-1 has additional binding partners other than IRAK1, and its function appears to be complex beyond involvement in innate and adaptive immune responses. Pellino-1 not only binds to RIP1 protein, a downstream protein of the adaptor TRIF protein (Chang *et al*, 2009), but also to the IKKε/TBK1 complex, which mediates TRIF-dependent IRF3 activation (Smith *et al*, 2011).

Pellino-1 knockout mice show resistance to LPS-induced endotoxin shock (Chang *et al*, 2009), abnormal T cell immune responses (Chang *et al*, 2011), and reduction in severity of experimental autoimmune encephalomyelitis (Xiao *et al*, 2013). These findings indicate that Pellino-1 is an important adaptor protein in TRIF-dependent TLR pathways and suggest that modulation of the Pellino-1 protein may be a valuable target to regulate TLR signaling. In addition, the TLR signaling cascade containing Pellino-1 is proposed to occur in the inner leaflet of membrane because the downstream signaling components, TAK1 and TAB 2, are part of the membrane complex (Moynagh, 2009).

This work was based on the idea that identification of the minimal region of Smad6 that binds to Pellino-1 and delivery to the inner leaflet of the membrane would allow the minimal region to bind to Pellino-1 and disrupt Pellino-1-mediated signaling complexes, resulting in therapeutic effects for TLR4-related inflammatory diseases.

We selected the sepsis model to validate our idea. Sepsis is a life-threatening disease characterized by severe systematic inflammation and multiple organ failure (Stearns-Kurosawa *et al*, 2011). Despite the critical importance of TLR signaling pathways in the pathogenesis of sepsis (Tsujimoto *et al*, 2008; Wittebole *et al*, 2010), recent clinical trials with eritoran (a synthetic inhibitor of MD2-TLR4) and TAK-242 (a small molecule inhibitor of TLR4 signaling) failed to improve the survival of patients with severe sepsis (Rice *et al*, 2010; Opal *et al*, 2013). However, due to the complexity of sepsis pathogenesis, it is yet early to rule out targeting specific component(s) of the TLR4 signaling pathway for sepsis treatment.

Protein–protein interactions are essential cellular processes through the formation of important regulatory signaling networks. Modulation of protein–protein interactions is gaining great interest as a new method to develop therapeutic molecules (Cochran, 2000; Wilson, 2009; Higueruelo *et al*, 2013). Pepducins are lipidated peptides attached to a palmitic acid to target the intracellular loops of G protein-coupled receptors (GPCRs) (Covic *et al*, 2002; Tressel *et al*, 2011). The attached lipid group partitions into the plasma membrane and flips across the membrane, delivering the conjugated peptide into the inner leaflet of the membrane. We applied this method to design the Smad6-derived peptide. We aimed to inhibit TLR4 signaling by attaching a palmitic acid to the minimal Pellino-1-binding region of Smad6 to deliver it to the inner leaflet of the cell membrane. Here, we demonstrate that this membrane-tethered Smad6-derived peptide, Smaducin-6, had therapeutic efficacy in mouse sepsis models: cecal-ligation–puncture (CLP)-induced and LPS-induced sepsis by inhibiting cytokine storm and apoptosis while enhancing neutrophil migration and bacterial clearance.

## Results

### Smad6 amino acids 422–441 inhibit TLR4 signaling

In addition to IRAKs (Grosshans *et al*, 1999; Jiang *et al*, 2003), Pellino-1 binds to other TLR4 signaling molecules, such as the RIP1 and IKKε/TBK1 complexes (Chang *et al*, 2009; Smith *et al*, 2011). Thus, disruption of Pellino-1-mediated signaling complexes may lead to profound inhibition of TLR4 signaling. Based on our previous report that TGF-β1-induced Smad6 was reported to disrupt IRAK1-mediated signaling complexes through direct binding to Pellino-1 (Choi *et al*, 2006), we first examined whether TGF-β1-induced Smad6 inhibits the formation of Pellino-1-mediated RIP1 and IKKε/TBK1 complexes. Immunoprecipitation assays showed that Smad6 disrupts these complexes through sequestering the Pellino-1 protein (Supplementary Fig S1). These results imply that Smad6 inhibits MyD88-dependent and MyD88-independent TLR4 pathways through targeting Pellino-1.

The major barrier of considering TGF-β signaling components for therapeutic application is that the TGF-β signaling pathway is pleiotropic and complex in terms of diverse cellular responses (Blobe *et al*, 2000) and thus may cause unexpected side effects. Smad6 encounters the same problems, as it has inhibitory effects targeting lipopolysaccharide (LPS)-induced pro-inflammatory NF-κB signaling (Choi *et al*, 2006) as well as being involved in TGF-β superfamily signaling through a negative feedback loop (Imamura *et al*, 1997; Inoue & Imamura, 2008). Therefore, we aimed to identify the minimal region of Smad6, which binds to Pellino-1 to specifically inhibit the LPS-induced pro-inflammatory signal.

**Figure 1:**
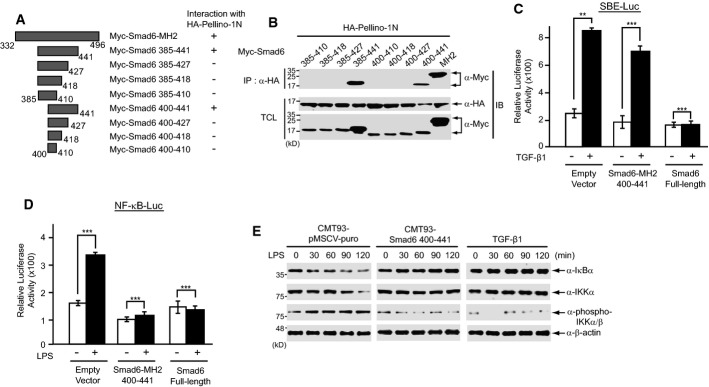
Amino acids 400–441 of Smad6 specifically bind to Pellino-1 and inhibit NF-κB signaling

We previously showed that the Smad6 MH2 domain (amino acids 332–496) is sufficient for interaction with Pellino-1N (amino acids 1–137) (Choi *et al*, 2006). To narrow down the subregion of the Smad6 MH2 domain responsible for binding to Pellino-1, serial truncations of the Smad6 MH2 domain were tested for interactions with Pellino-1 by coimmunoprecipitation assays (Fig1A and B). We found that Smad6 (amino acids 400–441) is sufficient for interaction with Pellino-1N, whereas Smad6 constructs with truncations of amino acids 410–441 failed to bind to the Pellino-1 N domain, suggesting that Smad6 (amino acids 410–441) is necessary for interaction with Pellino-1 (Fig1A and B). Interestingly, a subregion of the Smad6 MH2 domain (amino acids 400–441) was predicted to correspond to a β-sheet structure by homology modeling (Arnold *et al*, 2006) (Supplementary Fig S2).

A Schematic representation of truncated mutants of the Smad6 MH2 domain and binding to Pellino-1N. HA-tagged Pellino-1N encodes amino acids 1–137 of Pellino-1.

B Plasmids encoding truncated mutants of the Smad6 MH2 domain were co-transfected into HEK293 cells with HA-tagged Pellino-1N plasmid. Cell lysates were immunoprecipitated with anti-HA antibody and immunoblotted with anti-Myc antibody. Data are representative of at least three independent experiments. IP, immunoprecipitation; IB, immunoblot; TCL, total cell lysates.

C, D The SBE-Luc or 5xNF-κB-Luc reporter plasmid was co-transfected with an empty vector, Myc-Smad6 (400–441), or full-length Smad6-expressing plasmids into CMT-93 cells, respectively. After 24 h, cells were treated with TGF-β1 for 2 h or LPS for 2 h and luciferase activities were measured and normalized. Data were statistically analyzed by a *t*-test and show the mean ± SD of three independent experiments. **P < 0.05, ***P < 0.001.

E CMT-93 cell lines stably expressing Smad6 amino acids 400–441 were treated with LPS for the indicated time and expression of IκBα, IKKα, and phospho-IKKα/β was monitored by immunoblotting. As a positive control, CMT-93 cells were pre-treated with TGF-β1 for 2 h. CMT-93 cells stably expressing the empty vector pMSCV-puro were used as a negative control. β-actin was used as a loading control. All data are representative of three independent experiments. Source data are available online for this figure.

Because Pellino-1 was reported to be dispensable for IL-1R signaling in knockout mice (Chang *et al*, 2009), we examined whether the subregion of Smad6 (amino acids 400–441) specifically inhibits the LPS-induced TLR4 signal. Thus, we first measured LPS-induced NF-κB activity or TGF-β signaling activity using the 5xNF-κB-Luc reporter or SBE (Smad binding element)-Luc reporter, respectively, in RAW264.7 cells expressing Smad6 (amino acids 400–441), or full-length Smad6 plasmid as a control. Luciferase activity was measured following LPS or TGF-β1 treatment. Expression of full-length Smad6 inhibited both NF-κB-mediated and TGF-β-mediated reporter activity (Fig1C and D). In contrast, expression of the Smad6 β-sheet subregion (amino acids 400–441) inhibited only NF-κB-mediated reporter activity, and not TGF-β-mediated reporter activity (Fig1C and D). To further confirm the specific activity of this subregion in inhibiting the NF-κB signal, we generated stable CMT-93 epithelial cell lines expressing the Smad6 β-sheet region. CMT-93 cells are responsive to both TGF-β1 and LPS. Treatment with LPS induced IκBα degradation in control CMT-93 cells expressing an empty vector, whereas CMT-93 cells expressing the Smad6 β-sheet subregion did not show IκBα degradation upon LPS treatment, similar to the results of TGF-β1 pre-treatment for 2 h (Fig1E). These results indicate that the Pellino-1-binding subregion of Smad6 (amino acids 400–441) may be used to specifically modulate NF-κB-mediated pro-inflammatory signaling induced by LPS.

**Figure 2:**
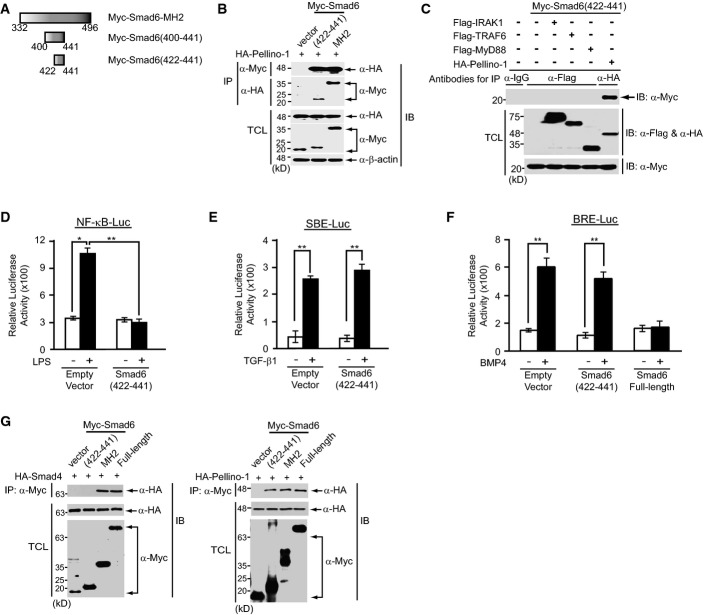
The minimal Pellino-1-binding region of Smad6 selectively inhibits NF-κB signaling

We sought to further narrow down this subregion of Smad6, composed of 42 amino acids, because a peptide of this length is limited in therapeutic usage *in vivo*. Co-immunoprecipitation assays showed that a minimal region of Smad6 amino acids 422–441 is sufficient for binding to HA-tagged Pellino-1 (Fig2A and B). This minimal region bound only to Pellino-1, but not to other proteins involved in the TLR4 signaling pathway (Fig2C). Expression of this minimal region of Smad6 (amino acids 422–441) inhibited NF-κB-mediated reporter activity, but not TGF-β or BMP-mediated reporter activity (Fig2D–F; Supplementary Fig S3). We next examined the binding of this minimal region to Smad4, because Smad6 MH2 domain interacts with Smad4 (Morén *et al*, 2005). Smad6 MH2 domain and full-length Smad6 bound to Smad4 and Pellino-1, respectively, whereas the minimal region of Smad6 (422–441) specifically bound to Pellino-1, but not to Smad4 (Fig2G). Thus, this minimal region of Smad6 (422–441) interacting with Pellino-1 specifically inhibits NF-κB activation.

A Schematic representation of truncated mutants of the Smad6 MH2 domain.

B A plasmid encoding the truncated mutant composed of Smad6 amino acids 422–441 (Myc-Smad6(422-441)) or wild-type Smad6 MH2 domain (Myc-Smad6-MH2) was co-transfected with the HA-tagged full-length Pellino-1 plasmid into HEK293 cells. Cell lysates were immunoprecipitated (IP) with anti-Myc or anti-HA antibody, and immunoblotted (IB) with anti-HA or anti-Myc antibody, respectively. The vector, CS3MTBXA-6xMyc, was transfected as a negative control. IP, immunoprecipitation; IB, immunoblot; TCL, total cell lysates. Data are representative of at least three independent experiments.

C The Myc-Smad6(422-441) plasmid was co-transfected with full-length Flag-IRAK1, Flag-TRAF6, Flag-MyD88, or HA-Pellino-1 plasmid into HEK293 cells. Cell lysates were immunoprecipitated with the indicated antibodies and immunoblotted with anti-Myc antibody. IgG was added as a negative control for IP. Data are representative of at least three independent experiments.

D, E The SBE-Luc or 5xNF-κB-Luc reporter plasmids were co-transfected with an empty vector or the Myc-Smad6(422-441) plasmid into CMT-93 cells, respectively. After 24 h, cells were treated with TGF-β1 for 6 h or LPS for 2 h, and luciferase activities were measured and normalized.

F The BRE-Luc reporter plasmid was co-transfected with an empty vector or the Myc-Smad6(422-441) plasmid or full-length Smad6 into RAW264.7 cells, respectively. After 24 h, cells were treated with BMP4 for 6 h and luciferase activity was measured and normalized.

G A plasmid encoding the truncated mutant composed of Smad6 amino acids 422–441 (Myc-Smad6(422-441)) or wild-type Smad6 MH2 domain (Myc-Smad6-MH2) or full-length Smad6 (Myc-Smad6) was co-transfected with HA-tagged full-length Smad4 or HA-tagged full-length Pellino-1 plasmid into HEK293 cells, respectively. Cell lysates were immunoprecipitated (IP) with anti-Myc and immunoblotted (IB) with anti-HA or anti-Myc antibody, respectively. The vector, pCS3MTBXA-6xMyc, was co-transfected with HA-Smad4 or HA-Pellino-1 as a negative control. Data are representative of at least three independent experiments. Data information: All data in (D–F) were statistically analyzed by a *t*-test and show the mean ± SD of three independent experiments. **P < 0.005, *P < 0.05 compared to the control (without LPS, TGF-β1, or BMP4). Source data are available online for this figure.

### Smaducin-6 inhibits the LPS-induced TLR4 signaling pathway

**Figure 3:**
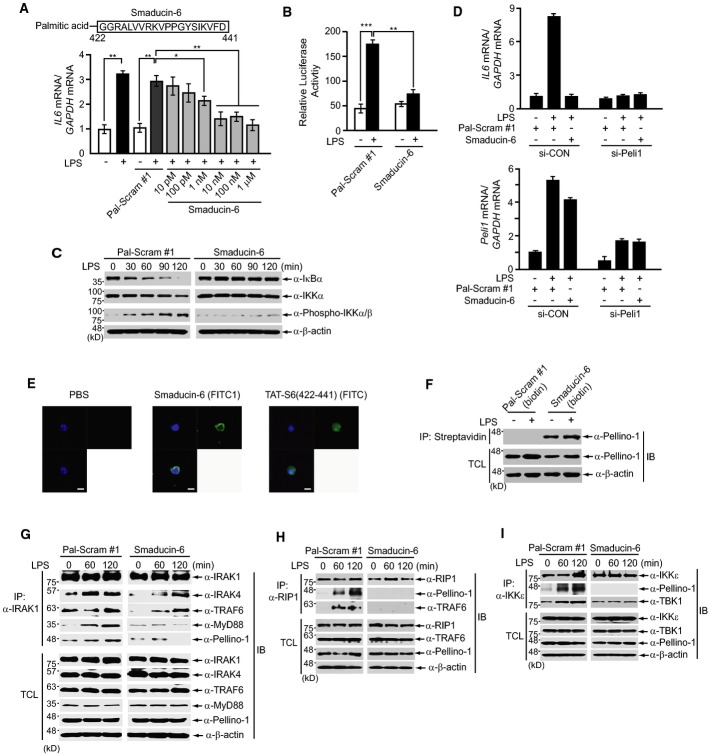
Membrane-tethered Smaducin-6 inhibits TLR4 signaling

Palmitoylated peptides have been reported to act as modulators, which target proteins at the intracellular surface of the membrane through a “flip” process (Covic *et al*, 2002; Tressel *et al*, 2011). We conjugated palmitic acid to the N-terminus of a commercially synthesized Smad6 (422–441) peptide, named Smaducin-6, to target Pellino-1 (Fig3A). Smaducin-6 was synthesized at a purity of more than 95% to be used in this study (Supplementary Fig S4A). Pre-treatment with Smaducin-6 reduced LPS-induced *Il6* gene expression in LPS-treated RAW264.7 cells in a dose-dependent manner (Fig3A), reduced NF-κB-mediated luciferase gene expression (Fig3B), and reduced IκBα degradation and IKKα/β phosphorylation (Fig3C). A scrambled palmitoylated peptide (Pal-Scram #1) was used as a negative control. In contrast, pre-treatment with Smaducin-6 did not affect LPS-induced phosphorylation of MAP kinases such as ERK, JNK, and p38 MPAK, compared to the control scrambled peptide (Supplementary Fig S4B). Also, the reduction in LPS-induced *Il6* gene expression by Smaducin-6 was similar to that of *Pellino-1* knockdown RAW264.7 cells (Fig3D).

A Pre-treatment of Smaducin-6 reduces LPS-induced interleukin-6 (*Il6*) gene expression in a dose-dependent manner in RAW264.7 cells. *Il6* gene expression was analyzed by quantitative real-time RT–PCR.

B, C Pre-treatment of 100 nM Smaducin-6 or scrambled peptide (Pal-Scram #1) for 30 min inhibits (B) NF-κB-mediated luciferase gene expression and (C) IκBα degradation and IKKα/β phosphorylation when RAW264.7 cells are treated with LPS for 2 h. Luciferase activity in (B) was normalized to β-galactosidase activity.

D *Peli1* knockdown or wild-type human THP1 cells were pre-treated with 100 nM Pal-Scram peptide and Smaducin-6 for 30 min and subsequently treated with LPS for 2 h. *Il6* and *Peli1* gene expression were analyzed by quantitative real-time RT–PCR. Data show the mean ± SD of three independent experiments.

E RAW264.7 cells were treated with 100 nM FITC-conjugated Smaducin-6 and TAT-S6(422-441), and localization was observed by confocal microscopy. Scale bar, 10 μm (magnification, 200×). Nuclei were stained with DAPI.

F After pre-treating RAW264.7 cells with biotin-conjugated Smaducin-6 (100 nM) for 30 min, cells were treated with LPS for 2 h. Subsequent precipitation by streptavidin–agarose showed that endogenous Pellino-1 binds to the Smaducin-6 peptide. A biotin-conjugated scrambled peptide was used as a negative control.

G–I Immunoprecipitation assays in primary peritoneal macrophages show that Smaducin-6 inhibits the formation of endogenous (G) IRAK1-, (H) RIP1-, or (I) IKKε-mediated signaling complexes induced by 2 h LPS treatment, compared to a scrambled peptide. IB, immunoblot; TCL, total cell lysates. Data shown are representative of three independent experiments. Data information: Data in (A) and (B) were statistically analyzed by a *t*-test and show the mean ± SD of three independent experiments. ***P < 0.001, **P < 0.005, *P < 0.05 compared to no LPS treatment or vehicle control (Pal-Scram #1). Source data are available online for this figure.

To confirm whether localization of Smaducin-6 to the intracellular surface of the plasma membrane is essential for inhibition of *Il6* gene expression, we generated FITC-conjugated Smaducin-6 and the same peptide conjugated with the cell-permeable HIV TAT protein transduction domain (Schwarze *et al*, 1999) (TAT-S6/422-441, Supplementary Table S1) and subsequently treated RAW264.7 cells with these peptides. FITC-conjugated Smaducin-6 inhibited LPS-induced *Il6* expression, but the TAT-S6/422-441 peptide did not (Supplementary Fig S5). Immunofluorescence analysis revealed that FITC-Smaducin-6 is mainly distributed in the membrane and cytoplasm (Fig3E), whereas the cell-permeable TAT-S6/422-441 peptide was localized in both the cytoplasm and nucleus (Fig3E). Streptavidin-mediated precipitation using biotinylated Smaducin-6 also showed a direct interaction between the Smaducin-6 peptide and endogenous Pellino-1 protein (Fig3F).

Because Pellino-1 is known to interact with the IRAK1, RIP1, or IKKε proteins (Jiang *et al*, 2003; Chang *et al*, 2009; Smith *et al*, 2011), we next investigated whether Smaducin-6 interferes with the formation of Pellino-1-mediated signaling complexes downstream of TLR4. Co-immunoprecipitation assays showed that Smaducin-6 inhibits the formation of IRAK1-, RIP1-, or IKKε-mediated signaling complexes induced by LPS treatment, compared to the scrambled peptide (Fig3G–I). We next examined whether the disruption of these signaling complexes by Smaducin-6 affects RIP1 kinase ubiquitination, because RIP1 ubiquitination by Pellino-1 is critical for the RIP1 downstream signaling pathway (Chang *et al*, 2009; Ofengein & Yuan, 2013). Plasmids encoding HA-tagged ubiquitin (Ubi), Flag-RIP1, and Flag-Pellino-1 were transiently transfected into HEK293 cells in the presence of Smaducin-6 or scrambled peptide. RIP1 polyubiquitination was examined under denaturing conditions of 1% SDS. Treatment of Smaducin-6 significantly inhibited RIP1 polyubiquitination induced by Pellino-1 (Supplementary Fig S6). These results indicated that Smaducin-6 binding to Pellino-1 inhibits Pellino-1 activity to ubiquitinate RIP1, resulting in the reduction in RIP1-mediated downstream signaling such as NF-κB. These findings suggest the therapeutic potential of Smaducin-6 on TLR4-related inflammatory diseases.

### Membrane-tethered Smaducin-6 protects mice from systemic sepsis

**Figure 4:**
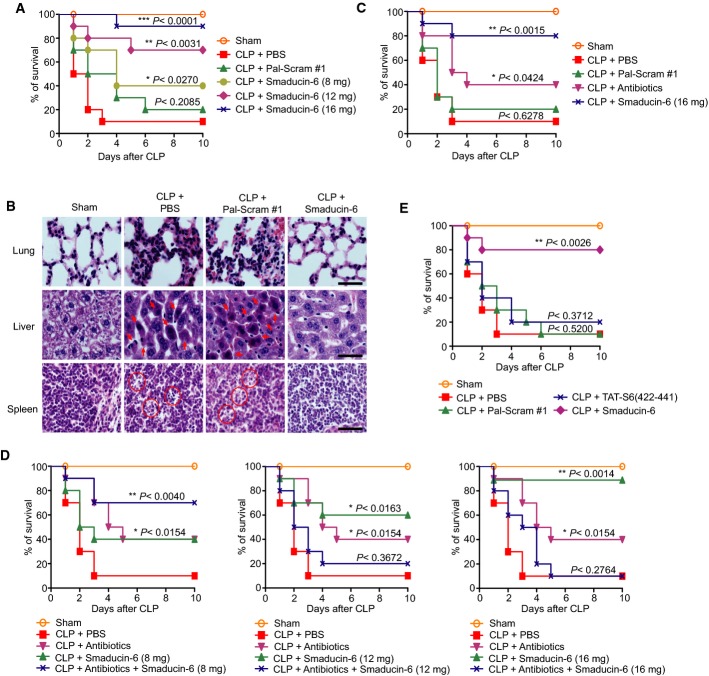
Smaducin-6 is therapeutic for mice with CLP-induced sepsis

To explore the therapeutic effect of Smaducin-6 on TLR4-related inflammatory diseases, we applied cecal-ligation–puncture (CLP) to BALB/c mice (Hubbard *et al*, 2005). We optimized the protocol for administration of Smaducin-6: time of initiation post-CLP and the route of injection. We subcutaneously injected total 16 mg/kg of Smaducin-6 into the mice subjected to CLP at 2, 6, and 10 h post-CLP followed by three injections at 12-h intervals. Initiation at 2 h post-CLP was the most effective with 90% survival rate, and later time points reduced the efficacy; 6 h post-CLP showed 60% survival rate, and 10 h post-CLP was ineffective (Supplementary Fig S7A). The best efficacy obtained at earlier time point is consistent with the fact that any anti-inflammatory therapies against sepsis should be initiated at early time point before developing septic shock, in which hemodynamic control is the most emergent and prioritized therapy. We then compared intravenous and subcutaneous injections at 2 h post-CLP followed by three injections at 12-h intervals. Intravenous injections of Smaducin-6 were not so effective as subcutaneous injections, showing 40–60% survival rates (Supplementary Fig S7B; Fig4A). We compared the tissue biodistributions of subcutaneously or intravenously injected Smaducin-6 in mice administered with fluorescent dye TAMRA (carboxytetramethylrhodamine)-conjugated Smaducin-6 (Supplementary Table S1). After 1 h of administration, we prepared various tissues from the mice and observed TAMRA using confocal microscope. Subcutaneously injected Smaducin-6 was distributed in the spleen, lungs, liver, and kidneys, whereas it was barely detected in the heart and brain (Supplementary Fig S8A). In contrast, intravenously injected Smaducin-6 was not detected as much as subcutaneously injected Smaducin-6 in any tested tissues (Supplementary Fig S8B). Subcutaneous injection is more effective than intravenous injection presumably because of the higher biodistribution of Smaducin-6 through lymphatic vessels than blood vessels. However, it is impossible to exclude other possibilities such as decreased stability of Smaducin-6 when intravenously injected.

Subcutaneous injection of Smaducin-6 increases the survival rate of severe CLP-induced sepsis mice (BALB/c mice). Different amounts of Smaducin-6 were subcutaneously injected at 2 h post-CLP followed by three injections at 12-h intervals. *n* = 10 mice per group per experiment.

Smaducin-6 treatment reduces abnormal nuclei and cell morphology observed in tissues of CLP-induced BALB/c mice, as observed by hematoxylin and eosin (H/E) staining. Data shown are representative of five independent experiments. Scale bar, 40 μm (magnification, 400×).

The survival rates of severe CLP-induced sepsis mice by subcutaneously injected Smaducin-6 were compared with those by intraperitoneally injected antibiotics (8 mg/kg gentamycin plus 8 mg/kg cephalosporin). Antibiotics were intraperitoneally injected into CLP mice at 2 h post-CLP followed by an additional injection after 12 h. *n* = 10 mice per group per experiment.

Therapeutic effects of Smaducin-6 alone, or antibiotics (8 mg/kg gentamycin plus 8 mg/kg cephalosporin), or different amounts of Smaducin-6 plus antibiotics were observed in CLP mice. Antibiotics were intraperitoneally injected into CLP mice at 2 h post-CLP followed by an additional injection after 12 h. Smaducin-6 was subcutaneously injected at 2 h post-CLP followed by three injections at 12-h intervals. *n* = 10 mice per group.

Comparison of mortality of severe CLP-induced sepsis mice upon subcutaneous injections of a total of 16 mg/kg Smaducin-6 or TAT-S6(422-441) peptide. *n* = 10 mice per group. Data information: In (A–E), PBS and scrambled peptide (Pal-Scram #1) were subcutaneously injected into CLP mice, respectively, as negative controls. Data in (A–E) were statistically analyzed by the log-rank test. ***P < 0.001, **P < 0.005, *P < 0.05 compared to vehicle control (CLP + Pal-Scram #1 or CLP + PBS).

We applied the most effective protocol with subcutaneous injections at 2 h post-CLP followed by three injections at 12-h intervals for further studies. Before doing further experiments, we generated two additional scrambled peptides (Pal-scram #2 and Pal-Scram #3) to exclude the possibility of non-specific effects of peptides and examined the binding of these peptides with Pellino-1 as well as the survival rates when these peptides were subcutaneously injected (Supplementary Fig S9A and B). Control groups treated with PBS or all scrambled peptides (Pal-Scram #1, Pal-scram #2 and Pal-Scram #3) developed severe sepsis with 90% mortality at 2–3 days, and the scrambled peptides did not bind to endogenous Pellino-1 (Fig4A; Supplementary Fig S9A and B). Thus, Pal-scram #1 was used as a control in subsequent experiments. By contrast, treatment with Smaducin-6 (16 mg/kg) dramatically reduced mortality to 10% with 90% survival rate (Fig4A). The therapeutic effect of Smaducin-6 was dose dependent; Smaducin-6 (12 mg/kg) resulted in 70% survival, and Smaducin-6 (8 mg/kg) resulted in 40% survival (Fig4A). Smaducin-6 also showed the similar therapeutic effect on CLP mice with less severe conditions (Supplementary Fig S9C). This therapeutic effect of Smaducin-6 was also observed in LPS-injected endotoxin shock mice (Supplementary Fig S10). Hematoxylin and eosin (H/E) staining in several types of tissue from CLP mice or the scrambled peptide-treated CLP mice revealed severe pulmonary inflammation with alveolar wall thickening, and necrosis of hepatocytes (arrow: increased eosinophilia of cytoplasm and pyknosis) and splenocytes (circle: karyorrhexis), whereas Smaducin-6 treatment significantly reduced these alterations (Fig4B).

Anti-sepsis therapies should be multifaceted targeting both pathogens and host immune responses. Therefore, we examined the synergistic effects of Smaducin-6 with antibiotics, which are commonly used for sepsis at clinical settings. Gentamycin (8 mg/kg) plus cephalosporin (8 mg/kg), whose spectra cover Gram-negative and Gram-positive bacteria, were administered to CLP mice. Antibiotics alone resulted in 40% survival rate, whereas Smaducin-6 (16 mg/kg) alone reduced mortality to 20% with 80% survival rate under the same condition (Fig4C). Low-dose Smaducin-6 (8 mg/kg) showed the synergistic effect on the survival improvement of CLP-induced mice to the similar level with Smaducin-6 (16 mg/kg) alone (Fig4D, left). In contrast, combination of high-dose Smaducin-6 (12 mg/kg and 16 mg/kg) and antibiotics completely abolished their therapeutic effects with the similar survival curve with the control group (Fig4D, middle and right). Although the mechanisms are unknown, these results show the critical importance of the dosing optimization for combination therapies with Smaducin-6 and antibiotics.

We designed Smaducin-6 to be tethered to the inner leaflet of the membrane by palmitic acid for biological efficacy. To confirm whether membrane tethering is essential for the therapeutic effect of Smaducin-6 on sepsis, we compared the effect of Smaducin-6 with that of the cell-permeable TAT-S6/442-441 peptide on the survival of CLP mice. When equal amount of each peptide (16 mg/kg) was subcutaneously injected into CLP mice, the TAT-S6/422-441 peptide did not protect mice from sepsis (Fig4E, blue line), although it was localized in the cytoplasm and nucleus (Fig3E). In contrast, Smaducin-6 showed 80% survival rate (Fig4E, purple line). Thus, tethering Smaducin-6 to the inner membrane by a palmitic acid is essential for its therapeutic effects.

### Smaducin-6 ameliorates excessive pro-inflammatory cytokine production in sepsis

**Figure 5:**
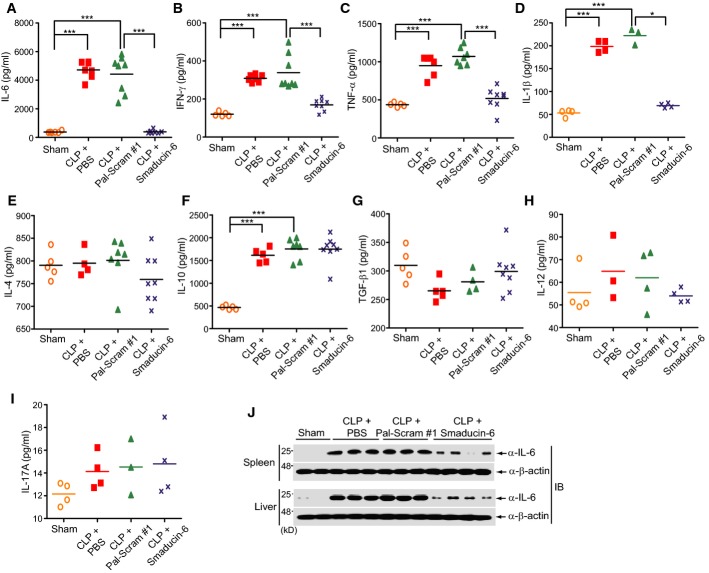
Smaducin-6 reduces systemic pro-inflammatory cytokines in CLP-induced sepsis mice

Cytokine storm is an important characteristic of sepsis, which results in multiple organ failure (Cavaillon *et al*, 2003). Treatment with Smaducin-6 significantly decreased the concentration of pro-inflammatory cytokines such as IL-6, IFN-γ, TNF-α, and IL-1β in both peripheral blood and peritoneal fluid of CLP mice after 18 h (Fig5A–D; Supplementary Figs S11A–D and S12). In contrast, the concentrations of IL-4, IL-10, TGF-β1, IL-12, and IL-17A did not show significant changes (Fig5E–I; Supplementary Fig S11E–I). Expression of IL-6 protein in the spleen and liver of Smaducin-6-treated CLP mice was significantly decreased compared with the control groups treated with PBS only or Pal-scram #1 (Fig5J). Reduction in pro-inflammatory cytokine levels in peripheral blood and peritoneal fluid explains the protective effect of Smaducin-6 on sepsis. Treatment with Smaducin-6 also reduced the level of CXCL2 chemokine, one of the hallmarks of sepsis (Supplementary Fig S11J).

A–I Smaducin-6 treatment decreased the concentration of IL-6, TNF-α, IFN-γ, and IL-1β in the blood of CLP mice (A–D), but not IL-4, IL-10, TGF-β1, IL-12, or IL-17A (E–I). Cytokine concentrations were analyzed by ELISA. *n* = 10 mice per group per experiment. Data were statistically analyzed by the Dunnett's multiple comparison test (one-way ANOVA). ***P < 0.001, **P < 0.005, *P < 0.05 compared to sham or vehicle control (CLP + Pal-Scram #1).

J Expression of the IL-6 protein is reduced in the spleen and liver of Smaducin-6-treated CLP mice. Each lane represents independent mice. Source data are available online for this figure.

### Smaducin-6 enhances bacterial clearance through neutrophil recruitment

**Figure 6:**
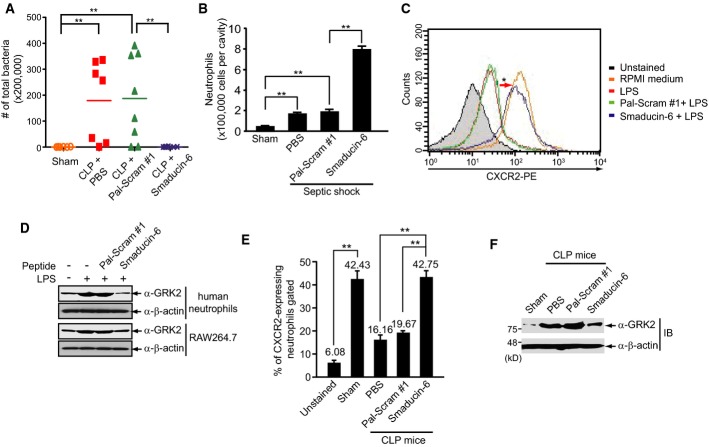
Smaducin-6 shows bactericidal effects through neutrophil recruitment

CLP-induced lethality is correlated with bacterial colony counts in peritoneal fluid (Cohen, 2002). Subcutaneous injection of Smaducin-6 into CLP mice dramatically decreased bacterial colony counts in both peritoneal fluid and blood (Fig6A; Supplementary Fig S13A). However, a direct killing effect of Smaducin-6 against bacteria was not observed (Supplementary Fig S13B). Since bactericidal effects in the sepsis model have been reported to be mediated mainly by neutrophil recruitment (Nathan, 2006), we examined whether Smaducin-6 increases neutrophil recruitment in an LPS-induced endotoxemia model. Smaducin-6 was subcutaneously administered 2 h after LPS injection into the peritoneum and injected again after 12 h. Numbers of neutrophils in collected peritoneal fluid was counted 24 h post-LPS injection. Treatment with Smaducin-6 significantly increased the numbers of recruited neutrophils in the peritoneal fluid, whereas treatment with the scrambled peptide did not (Fig6B).

Subcutaneous injection of Smaducin-6 into CLP mice reduced bacterial loads in peritoneal fluid. *n* = 10 mice per group per experiment. Data were statistically analyzed by the Mann–Whitney *U*-test. **P < 0.005 compared to sham or vehicle control (CLP + Pal-Scram #1).

Smaducin-6 increases neutrophil numbers in peritoneal lavage fluids from LPS-induced endotoxemia BALB/c mice. *n* = 5 mice per group per experiment. Data were statistically analyzed by a *t*-test. **P < 0.005 compared to sham or vehicle control (CLP + Pal-Scram #1).

CXCR2 expression in human neutrophils, measured by FACS, was decreased by LPS treatment and restored by Smaducin-6 treatment.

Treatment with Smaducin-6 downregulates LPS-induced GRK2 expression in RAW264.7 cells and human neutrophils.

CXCR2-expressing neutrophils isolated from CLP mice in the presence of Smaducin-6 or Pal-Scram peptide were analyzed by FACS. *n* = 5 mice per group were used. Data were statistically analyzed by a *t*-test and show the mean ± SD of at least three independent experiments. **P < 0.005 compared to sham or vehicle controls (CLP + PBS and CLP + Pal-Scram #1).

GRK2 expression was directly analyzed by immunoblotting in neutrophils isolated from CLP mice in the presence of Smaducin-6 or Pal-Scram #1 peptide. Data information: Data in (C), (D), and (F) are representative of three independent experiments. Source data are available online for this figure.

To determine how Smaducin-6 contributes to neutrophil recruitment, we examined the expression of the chemokine receptor CXCR2 and G protein-coupled receptor kinase-2 (GRK2) in human neutrophils in the presence of Smaducin-6. Decreased expression of CXCR2 in neutrophils has been associated with impaired neutrophil migration (Cummings *et al*, 1999; Vroon *et al*, 2006) and reported to be caused by TLR ligand-induced GRK2 expression (Loniewski *et al*, 2008; Alves-Filho *et al*, 2009). FACS analysis indicated that CXCR2 expression decreases upon LPS treatment of human neutrophils and is restored by Smaducin-6 treatment (Fig6C). Immunoblot analysis indicated that Smaducin-6 treatment down-regulates LPS-induced GRK2 expression in RAW264.7 cells and human neutrophils (Fig6D). These phenomena were also observed in neutrophils isolated from CLP mice treated with Smaducin-6 and scrambled peptides (Fig6E and F). These results show that treatment with Smaducin-6 restores neutrophil recruitment through re-expression of CXCR2 via reduction in GRK2 expression in neutrophils.

### Smaducin-6 significantly inhibits apoptosis in sepsis

**Figure 7:**
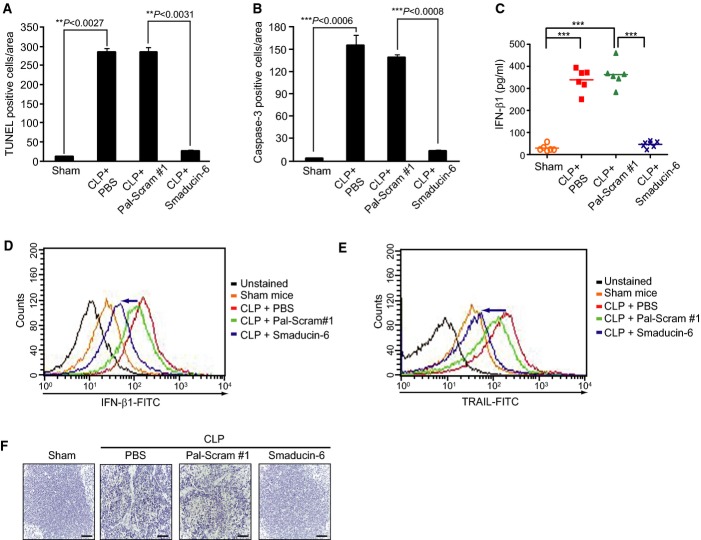
Smaducin-6 reduces sepsis-induced apoptosis

Extensive apoptosis of immune effector cells impairs immune responses in the pathogenesis of sepsis (Hotchkiss *et al*, 1999; Hotchkiss & Nicholson, 2006). In particular, rectification of the immunosuppressive state caused by extensive apoptosis has recently been shown as therapeutic for sepsis (Hotchkiss & Nicholson, 2006). Thus, we next examined the effects of Smaducin-6 peptide treatment on apoptosis in CLP-induced sepsis. Treatment with Smaducin-6 significantly reduced the numbers of TUNEL-positive cells in the spleens of CLP mice compared to the scrambled peptide-treated controls (Fig7A; Supplementary Fig S14A). Immunohistochemical analysis of caspase-3 expression also showed consistent results (Fig7B; Supplementary Fig S14B). Because the TRIF-mediated pathway via interaction with RIP1 has been reported to be more prominent in apoptosis than the MyD88-dependent pathway (Kaiser & Offermann, 2005), and Pellino-1 has been reported to bind to RIP (Chang *et al*, 2009), the inhibitory effect of Smaducin-6 on apoptosis in septic mice can be explained by disruption of the RIP1-mediated signaling complex (Fig3H).

A, B Quantitative analysis of (A) TUNEL-positive cells and (B) caspase-3-positive cells in IHC in the spleens of CLP + PBS, CLP + Pal-Scram #1, and CLP + Smaducin-6 mice. Three independent experiments (*n* = 3 mice per group per experiments) were performed. At least five hot spots in a section of TUNEL and IHC per experiment were selected, and average count was determined. The data were expressed as a mean percentage of total cell numbers and statistically analyzed by a *t*-test and show the mean ± SD of three independent experiments. **P < 0.005, ***P < 0.001 compared to sham or vehicle control (CLP + Pal-Scram #1).

C ELISA showing that Smaducin-6 treatment reduced IFN-β1 levels in peritoneal lavage fluids compared to Pal-Scram #1-treated CLP mice. *n* = 10 mice per group per experiment. Data were statistically analyzed by the Dunnett's multiple comparison test (one-way ANOVA). ***P < 0.001 compared to sham or vehicle control (CLP + Pal-Scram #1).

D, E IFN-β1 (D) and TRAIL (E) expression detected by FACS in splenocytes is decreased in Smaducin-6-treated CLP mice compared to scrambled peptide-treated CLP mice. Data are representative of three independent experiments.

F Decreased expression of TRAIL was detected by IHC in the spleen of CLP-induced mice treated with Smaducin-6 compared to mice treated with Pal-Scram. Scale bar, 100 μm (magnification, 200×). Data are representative of three independent experiments.

IFN-β1 is produced through the TRIF-dependent pathway by TLR4 or TLR3 activation (Takeuchi & Akira, 2010), and inhibition of IFN-β1 signaling reduces the mortality after CLP-induced sepsis (Dejager *et al*, 2014). Furthermore, IFN-β1 increases tumor necrosis factor (TNF)-related apoptosis-inducing ligand (TRAIL) in human peripheral blood T cells (Kayagaki *et al*, 1999). TRAIL has been recently found to be responsible for the sepsis-induced immuno-suppressive state. Apoptotic cells generated during sepsis induce TRAIL-producing CD8^+^ regulatory T cells, which subsequently suppress delayed-type hypersensitivity response (Unsinger *et al*, 2010). By contrast, *Trail*^−/−^ mice retain ability to mount antigen-specific CD8 T cell responses to secondary heterologous bacterial infection after sepsis induction by CLP model (Gurung *et al*, 2011). These findings indicate that TRAIL/TRAIL receptor pathway plays an important role in immune suppression in sepsis. Therefore, we examined whether Smaducin-6 decreases TRAIL expression by inhibiting IFN-β1 production in CLP-induced septic mice. ELISA showed that the levels of IFN-β1 in peritoneal lavage fluids of Smaducin-6-treated CLP mice are significantly decreased compared with those of Pal-Scram #1-treated CLP mice (Fig7C). Consistently, FACS analyses showed that expression of IFN-β1 is also decreased in the splenocytes of Smaducin-6-treated CLP mice compared with that of Pal-Scram #1-treated CLP mice (Fig7D). In accordance with the levels of IFN-β1, expression of TRAIL in the spleens determined by FACS and immunohistochemistry was decreased in Smaducin-6-treated CLP mice (Fig7E and F). Disruption of the IKKε/TBK1/Pellino-1 and/or RIR1/Pellino-1 complexes by Smaducin-6 (Fig3H and I) accounts for the reduction in IFN-β1 and TRAIL in immune cells.

## Discussion

Because overwhelming activation of TLR4 signaling underlies the pathogenesis of sepsis (Weighardt & Holzmann, 2007; Tsujimoto *et al*, 2008), various TLR4 antagonists have been developed and some have entered clinical trials (Tsujimoto *et al*, 2008; Rice *et al*, 2010; Wittebole *et al*, 2010; Opal *et al*, 2013). Failure in the recent clinical trial of TAK-242, a small molecule inhibitor of TLR4, and that of eritoran, a specific antagonist of MD2-TLR4, for patients with severe sepsis have cast a shadow on the idea to target TLR4 to develop novel therapeutic interventions for sepsis. However, it is yet early to exclude targeting the TLR4 pathway for sepsis. The phase 3 trial with eritoran particularly had major caveats, including heterogeneity of serum endotoxin levels in the enrolled patients, a lower mortality rate in the placebo group than anticipated, and differential outcomes of patients infected with Gram-positive bacteria between the phase 2 and phase 3 trial (Opal *et al*, 2013). Moreover, both TAK-242 and eritoran inhibit all the signaling pathways downstream of TLR4. It is highly possible that the specific targeting of the crucial mediator of TLR4 downstream pathway is therapeutic. Therefore, identification of such signaling molecules would lead to the development of therapeutic interventions to specifically target them.

Although TGF-β and its signaling protein Smad6 are involved in anti-inflammatory responses through disruption of Pellino-1-mediated TLR4 signaling complex (Choi *et al*, 2006) and selective degradation of MyD88 (Lee *et al*, 2011), therapeutic interventions based on these signaling components have not been attempted for TLR4-related inflammatory diseases. This study is the first report demonstrating the therapeutic potential of a Smad6-derived peptide (Smaducin-6) in sepsis treatment.

To enforce the protein–protein interaction between Smad6 and membrane-associated Pellino-1 to disrupt the formation of Pellino-1-mediated TLR4 signaling complex, we have decided to apply the technique to generate cell-penetrating membrane-tethered palmitoylated peptides, which have been reported to selectively modulate the activity of G protein-coupled receptors (GPCRs) (Covic *et al*, 2002; Tressel *et al*, 2011). Therapeutic ineffectiveness of a cell-permeable TAT-conjugated peptide (TAT-S6/422-441) (Fig4E) indicates that membrane tethering by palmitic acid is crucial for the therapeutic effects of Smaducin-6 on sepsis. Several groups have also shown that peptides mimicking specific sequences conjugated to palmitic acid are useful in modulating specific pathways (Cisowski *et al*, 2011; Valente *et al*, 2011). Because Smaducin-6 is able to interfere with protein–protein interactions of the inflammatory TLR4 signaling cascade through targeting a membrane-associated Pellino-1 protein, our findings strongly suggest that a well-designed palmitoylated peptide can be used to antagonize specific signaling proteins located in the inner leaflet of the cell membrane and to mediate negative cross talk between different signaling pathways.

Recent advances have revealed the importance of apoptosis of immune cells in addition to hyper-inflammation in the pathogenesis of sepsis (Hotchkiss & Nicholson, 2006). Therefore, Pellino-1 is a valuable target because it is involved in both apoptosis and hyper-inflammation and apoptosis pathways through interactions with various proteins such as RIP1, IKKε, and IRKA1 (Jiang *et al*, 2003; Chang *et al*, 2009; Smith *et al*, 2011). Indeed, we found that Smaducin-6 directly binds to Pellino-1 and disrupts three important signaling complexes in the TLR4 pathway, eventually resulting in the inhibition of pro-inflammatory cytokines as well as apoptosis in sepsis models.

RIP1 is a key regulator that controls both inflammatory signaling and cell death. We found that disruption of Pellino-1-mediated signaling complexes by Smaducin-6 inhibits RIP1 polyubiquitination, which is critical for RIP1 downstream signaling pathways that leads to NF-κB activation and IFN-β1 production (Fig3H; Supplementary Fig S6). However, Smaducin-6 did not inhibit IRAK1 phosphorylation although it disrupted the LPS-induced IRAK1-Pellino-1 signaling complex (Supplementary Fig S15). Although phosphorylation of Pellino-1 by IRAK1 increases polyubiquitinations of IRAK1 and Pellno-1 itself in *in vitro* system (Butler *et al*, 2007; Ordureau *et al*, 2008), relevance of phosphorylation and ubiquitination of Pellino-1 and IRAK1 has not been verified in *in vivo*. Thus, our findings that the disruption of IRAK1-mediated signaling complex by Smaducin-6 did not affect IRAK1 phosphorylation suggest the presence of an unidentified enzyme independent of Pellino-1, which regulates IRAK1 phosphorylation.

Upregulation of TRAIL by IFN-β1 is also crucial for apoptosis of immune cells in sepsis (Unsinger *et al*, 2010; Gurung *et al*, 2011). Our results indicate a correlation between IFN-β1 and TRAIL expression in mouse splenocytes upon LPS treatment in the presence or absence of Smaducin-6 (Fig7C–F). A number of reports indicate new physiological roles of TRAIL beyond its traditional role as an apoptosis inducer in cancer cells (Diehl *et al*, 2004; Corazza *et al*, 2009), and increased attention has recently been paid to the functions of TRAIL in sepsis (Unsinger *et al*, 2010; Gurung *et al*, 2011). Furthermore, administration of a neutralizing anti-TRAIL mono-clonal antibody to CLP-treated wild-type mice restored CD8^+^ T cell immunity (Gurung *et al*, 2011). Based on these findings together with our results, it is possible that TLR3 or TLR4 activation increases IFN-β1, which subsequently increases TRAIL in CD8^+^ regulatory T cells during sepsis, finally contributing to the immunosuppressive state of sepsis.

In addition, we demonstrate that Smaducin-6 enhances neutrophil recruitment by restoring the expression of CXCR2, which is reduced by TLR ligand-induced GRK2. Although the results that Smaducin-6 restored LPS-induced CXCR2 reduction are similar with those recently obtained upon IL-33 treatment in sepsis (Alves-Filho *et al*, 2010), the mechanism may be different. IL-33 increased CXCR2 expression by downregulation of GRK2 through the receptor complex ST2-IL-1RAP (Alves-Filho *et al*, 2010). In contrast, TLR ligands induce GRK2 expression, subsequently downregulating CXCR2 expression in neutrophils (Cummings *et al*, 1999; Vroon *et al*, 2006; Loniewski *et al*, 2008; Alves-Filho *et al*, 2009). Based on these previous findings and our present results, it is likely that Smaducin-6 decreases LPS-induced GRK2 expression presumably through direct binding to Pellino-1, resulting in the restoration of CXCR2 expression for neutrophil migration.

Although we reveal the molecular mechanisms of the therapeutic effects Smaducin-6 has on sepsis, we do not claim that the present form of the Smaducin-6 peptide is directly applicable to sepsis treatment. Future detailed optimization studies for developing the optimal administration protocol are required before clinical application of Smaducin-6 to sepsis. First, pharmacokinetics and pharmacodynamics of Smaducin-6 should be determined, although we show that Smaducin-6 was mainly distributed in the spleen, lungs, liver, and kidney (Supplementary Fig S8). The pharmacological activity of Smaducin-6 might be inferred from the pepducin P4pal-10, which is a well-known protease-activated receptor 4 (PAR4) i3 loop-based pepducin composed of 10 amino acids (Covic *et al*, 2007; Tressel *et al*, 2011). P4pal-10 shows a half-life of 3.5 h when intravenously injected into mice and shows a prolonged half-life of approximately 14 h when subcutaneously injected (Covic *et al*, 2007). P4pal-10 pepducin subcutaneously injected at a dose of 10 mg/kg is distributed in a variety of tissues except for the brain (Tressel *et al*, 2011). Tissue distributions of Smaducin-6 and the more efficacy of Smaducin-6 obtained by subcutaneous injection than intravenous injection were similar to P4pal-10, which implicates that the delivery of palmitoylated peptides *in vivo* might be more efficient through lymphatic vessels compared with blood vessels. It might correlate with the dominant distributions of Smaducin-6 to lymphatic-vessel-rich organs, such as lung, spleen, and liver (Supplementary Fig S8). Although systemically administered palmitoylated peptides are distributed to highly vascularized tissues (Tressel *et al*, 2011), similar to Smaducin-6, little is known about which cell types within tissues of animal models are targeted by palmitoylated peptides and how these peptides reach the target cells. We speculate that Smaducin-6 shows the highest efficacy in innate immune cells residing in the lymphatic-vessel-rich organs, in which Pellino-1-mediated TLR4 signaling plays the most important pathophysiological roles in sepsis.

Our findings also clearly reveal the critical importance of optimization for the combination therapies of Smaducin-6 with various antibiotics for sepsis. Combination of antibiotics with low-dose Smaducin-6 (8 mg/kg) showed the synergistic effect, whereas that with high-dose Smaducin-6 (12 and 16 mg/kg) completely canceled out their therapeutic effects each other (Fig4D). Among the bacteriocidal β-lactam antibiotics, cephalosporin induces release of high levels of endotoxin and other components from Gram-negative bacteria (Kirikae *et al*, 1997). When exposed to higher amounts of endotoxins by the treatment with cephalosporin, it might be possible that profound inhibition of Pellino-1-mediated cascade by high-dose Smaducin-6 upregulates other Pellino-1-independent TLR4 inflammatory signaling cascades, whereas partial inhibition of Pellino-1-mediated cascade by low-dose Smaducin-6 synergistically works with bactericidal antibiotics. Combination of Smaducin-6 with other β-lactam antibiotics, such as imipenem, which do not induce the release of endotoxin with more appropriate spectra covering Gram-negative bacteria, should be optimized in the future studies (Kirikae *et al*, 1997).

Another interesting finding in this study is that the minimal region of Smad6 (amino acids 422–441) that binds to Pellino-1 can also bind to Pellino-3, but not Pellino-2, although these proteins show considerable homology (Supplementary Fig S16). Since Pellino-3 is known to be a negative feedback component in the TLR3 pathway (Siednienko *et al*, 2012), a mediator of NOD2 signaling (Yang *et al*, 2013a), and a regulator of TNF-α signaling (Yang *et al*, 2013b), this result suggests that Smaducin-6 may also regulate Pellino-3-mediated signaling pathways. However, to test this possibility, we first need to test whether Pellino-3 can compensate for Pellino-1 function in a Pellino-1 deficiency and whether Pellino-3 has a unique role in cellular signaling compared with Pellino-1, which is beyond the scope of this study.

Smaducin-6 may be applicable to other inflammatory diseases caused by abnormal TLR4 activation because Pellino-1 acts as an important platform of the TLR4 signaling pathway. Our results indicate that Smad6 amino acids 422–441 composing Smaducin-6 specifically bind to Pellino-1N (amino acids 1–137), which contains several forkhead-associated (FHA) domains (Lin *et al*, 2008; Moynagh, 2014). Therefore, our results imply that Smaducin-6 interacts with a Pellino-1 FHA domain. The design of chemicals mimicking the interaction of Smaducin-6 with a Pellino-1 FHA domain, as well as structural analysis of the binding between Smaducin-6 and Pellino-1, may be helpful to develop drugs for TLR4-related inflammatory diseases.

## Materials and Methods

### Study approval

Animal experiments were approved by the Institutional Animal Care and Use Committee at the Department of Biological Sciences, Sungkyunkwan University (Suwon, Korea). The isolation of human neutrophils was approved by the Institutional Animal Care and Use Committee at Ajou University College of Medicine (Suwon, Korea). Animals were housed in a pathogen-free barrier facility with a 12-h light/dark cycles and maintained on standard chow. Animal care and all experimental procedures were conducted in accordance with the Guide for Animal Experiments published by the Korea Academy of Medical Sciences and the safety guidelines of Sungkyukwan University.

### Peptides

Palmitic acid-conjugated Smad6 amino acids 422–441 (Smaducin-6), palmitic acid-conjugated scrambled Smad6 peptides (Pal-Scram; negative control), and FITC-, biotin-, TAMRA-conjugated peptides were commercially synthesized and purified by Anygen (Korea). The amino acid sequences of the peptides in this study are described in Supplementary Table S1.

### Plasmids

Full-length Pellino-1 cDNA was cloned into the *Eco*RI and *Bam*HI sites of the pSG5-2xHA vector or the *Eco*R1 and *Xho*I sites of the pcDNA3-Flag vector, resulting in HA-Pellino-1 or Flag-Pellino-1, respectively. The plasmid encoding amino acids 1–140 of the Pellino-1 protein (HA-Pellino-1N) was previously described (Choi *et al*, 2006). Plasmids encoding different regions of the Smad6 protein (Myc-Smad6 MH2, Myc-Smad6 346F, Myc-Smad6 371F, Myc-Smad6 385F, Myc-Smad6 464R, Myc-Smad6 441R, Myc-Smad6 410R, Myc-Smad6 385-441, Myc-Smad6 385-427, Myc-Smad6 385-418, Myc-Smad6 385-410, Myc-Smad6 400-441, Myc-Smad6 400-427, Myc-Smad6 400-418, Myc-Smad6 400-410, Myc-Smad6 422-441, Myc-Smad6) were amplified from full-length Smad6 cDNA by PCR and subcloned into the *Bam*HI and *Xho*I sites of the pCS3MTBXA-6xMyc vector. The full-length Pellino-2, Pellino-3a, and Pellino-3b cDNA were amplified from plasmids kindly provided by Dr. Suntaek Hong (Gachon University, Korea) and subcloned into the *Eco*RI and *Xho*I sites or *Eco*RI and *Sal*I of the pcDNA3-HA vector, respectively. The plasmid encoding HA-Smad4 was kindly provided by Dr. Byung-Chul Kim (Kangwon University, Korea). Sequences of the PCR-generated portions of all constructs were verified by sequencing. Primer sequences are described in Supplementary Table S2. The 5xNF-κB-Luc luciferase reporter plasmid was purchased from Clontech Laboratories. The SBE-Luc and BRE-Luc luciferase reporter plasmids were previously described, respectively (Ishida *et al*, 2000; Kim *et al*, 2004). Plasmids encoding Flag-RIP1 and HA-Ubiquitin (Ubi) were kindly provided by Dr. Jaewhan Song (Yonsei University, Korea).

### Reagents, cell culture, and primary peritoneal macrophages

Recombinant human TGF-β1 and recombinant IL-1β were obtained from R&D Systems. LPS (*Escherichia coli* serotype O11:B4) was purchased from Sigma. CMT-93 murine intestinal epithelial cells, human embryonic kidney 293 (HEK293) cells, and RAW264.7 macrophage cells were maintained in DMEM with 10% FBS (GIBCO-BRL). Human monocyte THP1 cells were maintained in RPMI with 10% PBS. All cell lines used in this study were purchased from American Type Culture Collection (Manassas, VA, USA). Gentamycin and cephalosporin were kindly provided from Ajou University College of Medicine. The isolation of peritoneal macrophages from 7-week-old C57BL/6 female mice (Daehan biolink, Korea) was performed as previously described (Choi *et al*, 2006).

### Antibodies, immunoblot, immunoprecipitation, and ubiquitination assays

Mouse anti-HA (F-7, dilution ratio 1:1,000), mouse anti-c-Myc (9E10, 1:1,000), rabbit anti-IκBα (C-21, 1:1,000), mouse anti-IRAK1 (F-4, 1:1,000), and mouse anti-TRAF6 (D-10, 1:1,000) were purchased from Santa Cruz Biotechnology. Antibody for rabbit anti-Pellino-1 (1:1,000) was kindly provided by Dr. Peter C.F. Cheung (Nanyang Technological University, Singapore). Antibody for rabbit anti-IRAK4 (1:1,000) was purchased from Imgenex. Rabbit anti-Smad6 (1:1,000), rabbit anti-MyD88 (D80F5, 1:1,000), rabbit anti-IKKα (1;1,000), rabbit anti-phospho-IKKα/β (1:1,000), rabbit anti-RIP1 (D94C12, 1:1,000), rabbit anti-phospho-IRAK1 (T209, 1:1,000), rabbit anti-IKKε (anti-IKKi; D61F9, 1:1,000), rabbit anti-TBK1 (D1B4, 1:1,000), and rabbit anti-GRK2 (1:1,000) were purchased from Cell Signaling. Anti-β-actin antibody (1:5,000) was obtained from Sigma. Cells were transfected with the indicated plasmids or pre-treated with peptides under certain conditions, and subsequently treated with LPS. Cells were harvested and lysed with lysis buffer (PBS containing 0.5% Triton X-100, 20 mM HEPES (pH 7.4), 150 mM NaCl, 12.5 mM β-glycerol phosphate, 1.5 mM MgCl_2_, 10 mM NaF, 2 mM DTT, 1 mM NaOV, 2 mM EGTA, 1 mM PMSF, and protease inhibitor cocktail). The samples were cleared by centrifugation at 13,000 rpm for 10 min. For immunoprecipitation, cell lysates were incubated with protein A/G agarose beads and with indicated antibodies at 4°C for 12 h. The beads were washed three times with lysis buffer, and immunoprecipitates were separated from the beads by adding 2× sample buffer and boiled and fractioned by SDS–PAGE. Immunoblot analysis was subsequently performed using the indicated antibodies. After pre-treatment of 100 nM Pal-Scram #1 or Smaducin-6 for 2 h, plasmids encoding HA-tagged ubiquitin (Ubi), Flag-RIP1, and Flag-Pellino-1 were transiently transfected into HEK293 cells. After 24 h, cells were harvested to examine the polyubiquitination of RIP1 protein. The ubiquitination assay for RIP1 protein was performed as previously described (Jung *et al*, 2013).

### Sepsis models

Cecal ligation and puncture (CLP)-induced sepsis mice were prepared from 6-week-old BALB/c male mice (Daehan biolink, Korea) as previously described (Kim *et al*, 2010). Sham mice were subjected to the same procedure, but without puncture of the cecum. For LPS-induced endotoxemia model, 60 mg/kg of LPS was intraperitoneally injected as previously described (Kim *et al*, 2010). After 2 h, 100 μg/kg of peptide was injected subcutaneously or intravenously four times at 12-h intervals (total 16 mg/kg). The survival rate was monitored daily for 10 days.

### Cytokine and chemokine measurements

To measure the secretion of CLP-induced cytokines or chemokines in peritoneal lavage fluids or blood, mice were given peptides 2 and 14 h after CLP. Samples were collected at 24 h after CLP, and concentrations of cytokines and chemokines were determined by ELISA using various antibodies. Mouse IL-6 (88-7064), IFN-γ (88-7314), TNF-α (88-7346), IL-4 (88-7044), IL-10 (88-7104), TGF-β1 (88-7344), IL-12 p70 (88-7121), and IL-17A (88-7371) ELISA Ready-SET-GO were purchased from eBioscience. The Mouse IL-1β ELISA Set (559603) was purchased from BD Sciences. The Mouse CXCL2/MIP-2 immunoassay (MM200) was purchased from R&D Systems. The Mouse IFN-β ELISA Kit was purchased from PBL Biomedical Laboratories. Values were calculated on the basis of a standard curve constructed for each assay. The data show the mean value ± SD of three independent experiments.

### Tissue protein extraction

After CLP surgery, isolated spleen and liver were frozen with liquid nitrogen and ground by a homogenizer. Samples were washed with PBS and centrifuged at 13,000 rpm for 10 min. Cells were lysed with lysis buffer [10 mM Tris (pH 7.4), 150 mM NaCl, 1% Triton X-100 1 mM EDTA, 1 mM EGTA, 0.2 mM NaOV, 0.5% NP-40, protease inhibitor cocktail] and sonicated. Samples were cleared by centrifugation at 13,000 rpm for 10 min and used for immunoblot analysis.

**The paper explained**

**Problem**

Mortality of Gram-negative bacterial sepsis caused by hyperactivation of TLR4 signaling pathway remains extremely high despite the vigorous investigation to develop new therapeutic interventions targeting both pathogens and host immune responses. Recent findings including ours have revealed that Smad6, one of the inhibitory Smads of TGF-β/BMP signaling pathway, binds to Pellino-1, thereby suppressing TLR4 pro-inflammatory signal. Thus, the interaction between Smad6 and Pellino-1 can be a therapeutic target for sepsis. However, maneuvers to target this protein–protein interaction remain yet unestablished.

**Results**

Here, we synthesized a membrane-tethered palmitic acid-conjugated peptide composed of 20 amino acids 422–441 of Smad6, named Smaducin-6. Smaducin-6 specifically bound to Pellino-1 protein, which then disrupted Pellino-1-involved RIP1-, IKKε- or IRAK1-mediated TLR4 signaling complexes. We found that Smaducin-6 had a significant therapeutic efficacy in mouse sepsis models: cecal-ligation–puncture (CLP)-induced and LPS-induced sepsis. Disruption of hyperactivated TLR4 signaling complexes by Smaducin-6 significantly improved survival of sepsis by inhibiting pro-inflammatory cytokine production and apoptosis, and by enhancing neutrophil migration and bacterial clearance.

**Impact**

This study is the first report demonstrating the potent efficacy of Smaducin-6: a membrane-tethered peptide mimicking protein–protein interaction between Smad6 and Pellino-1 in sepsis. Failures of phase 3 clinical trials of eritoran, a TLR4 antagonist, and TAK242, a small molecule inhibitor of TLR4, indicate that TLR4 itself could not be a therapeutic target. Our findings show that specific targeting of the TLR4 downstream signaling pathway via direct interaction of Smaducin-6 peptide with Pellino-1 can be the novel and effective peptide-based therapeutic intervention for sepsis.

### Neutrophil and splenocyte isolation

Mouse neutrophils were isolated from the peritoneal cavities of LPS-induced endotoxin shock or CLP mice using the Histopaque-1077 solution (Sigma). Cell counts were carried out using Trypan Blue (Sigma). Human neutrophils were isolated from the peripheral blood of a healthy human donor using the Histopaque-1077 solution (Sigma) through a density gradient. The peripheral bloods were collected with written informed consent, which was approved by the Institutional Animal Care and Use Committee at Ajou University College of Medicine (Suwon, Korea). Mouse splenocytes were isolated from the spleen after CLP. Isolated spleens were ground using the Tissue Sieve System (Bellco Glass). Ground cells were filtrated by a 70-um Cell Strainer (BD Falcon), and blood cells were lysed by RBC lysis buffer. Splenocytes were gained through washing with PBS three times.

### Statistical analysis

Statistical significance was calculated by using the GraphPad Prism 5 Software (GraphPad Software). Survival studies were analyzed with the log-rank test, and bacteria and neutrophils counts were analyzed by the Mann–Whitney *U*-test. ELISA studies were analyzed by the Dunnett's multiple comparison test (one-way ANOVA). A *t*-test (unpaired to test with Welch's correction) was used to compare the differences between two groups. *P* < 0.05 was considered statistically significant. The variance similar between the groups was confirmed by Levene's test.

Details of the bacterial counts, flow cytometry analysis, immunohistochemsitry, immunofluorescence, modeling of Smad6 MH2 domain, transient transfection, reporter assay, *Peli1* knockdown, RNA extraction, and quantitative real-time RT–PCR are provided in the Supplementary Information.

## Supporting Information

Source Data for Supplementary Figure S16

Review Process File
