# Supplementary material for: Inhibition of lethal inflammatory responses through the targeting of membrane-associated Toll-like receptor 4 signaling complexes with a Smad6-derived peptide
Source: EMBO Mol Med. 2015 Mar 12;7(5):577–92. doi: 10.15252/emmm.201404653 (PMC4492818; doi:10.15252/emmm.201404653)
Supplement: Supplementary file 1 [file emmm0007-0577-sd1.pdf]

Supplementary Information

Inhibition of lethal inflammatory responses through the targeting of membrane-associated Toll-like receptor 4 signaling complexes with a Smad6-derived peptide

Youn Sook Lee, Jin Seok Park, Su Myung Jung, Sang-Doo Kim, Jun Hwan Kim, Jae Young Lee, Kyeong Cheon Jung, Mizuko Mamura, Sangho Lee, Seong-Jin Kim, Yoe-Sik Bae, Seok Hee Park

Inventory of Supplementary Information

16 Supplementary figures

2 Supplementary Tables

Supplementary materials and methods

<Supplementary Figures>

Supplementary Fig.S1

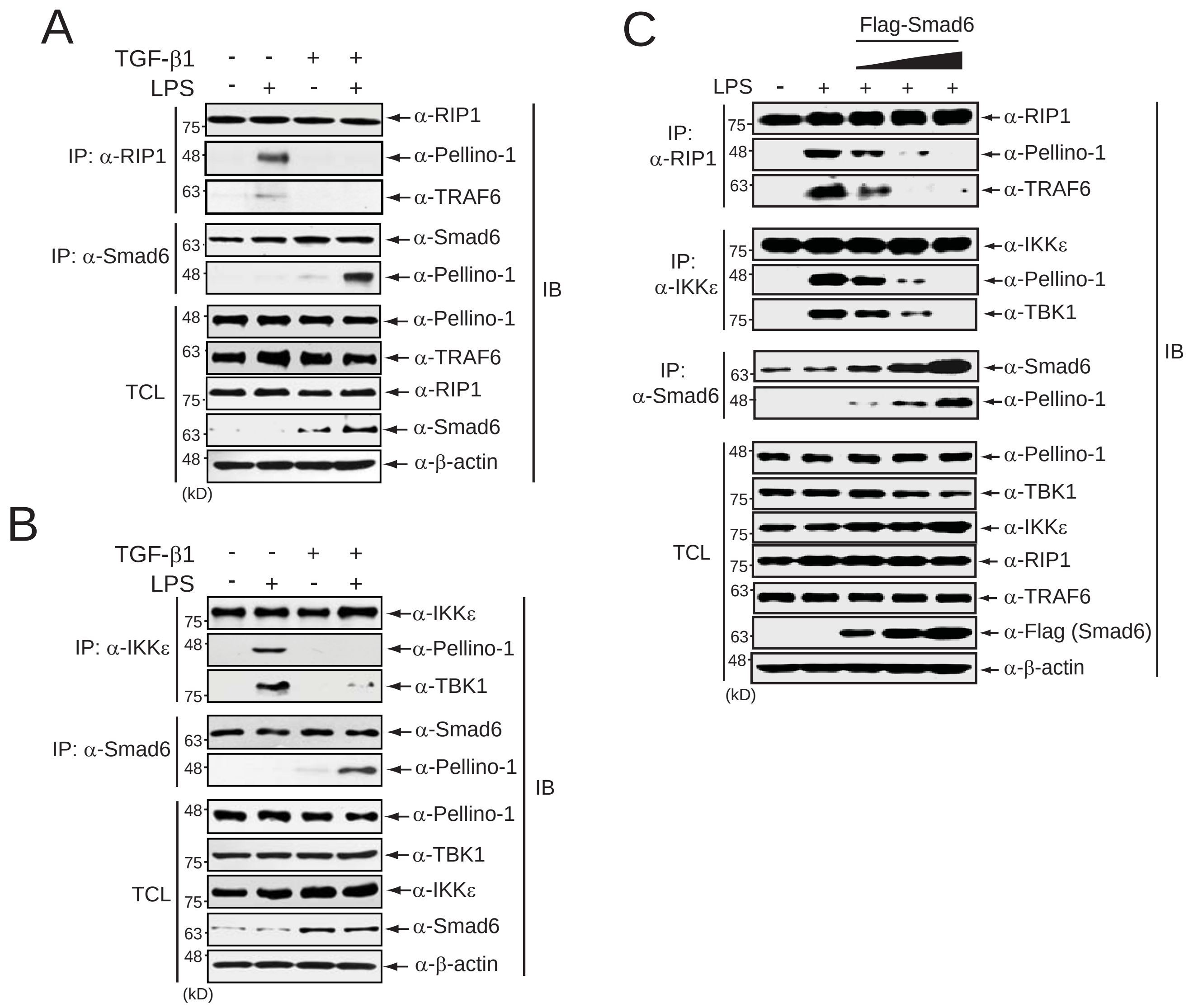

Supplementary Fig. S1. TGF-β1-induced Smad6 disrupts RIP1-mediated and IKKε-mediated signaling complexes via sequestering Pellino-1.
A, B. Primary peritoneal macrophages were pre-treated with TGF-β1 for 2 h and subsequently treated with LPS for 2 h. As controls, cells were treated with only LPS or TGF-β1. Cell lysates were immunoprecipitated with (**A**) anti-RIP1 antibody or (**B**) anti-IKKε antibody and in turn immunoblotted with the indicated antibodies against endogenous proteins. **C**. A plasmid encoding full-length Smad6 was transfected into RAW264.7 cells in a dose-dependent manner. After 24 h, cells were treated with LPS for 2 h. Cell lysates were immunoprecipitated with anti-RIP1, anti-IKKε and anti-Smad6 antibodies against endogenous proteins, respectively, and subsequently immunoblotted with the indicated antibodies. Data are representative of at least three independent experiments. IP, immunoprecipitation; IB, immunoblot; TCL, total cell lysates.

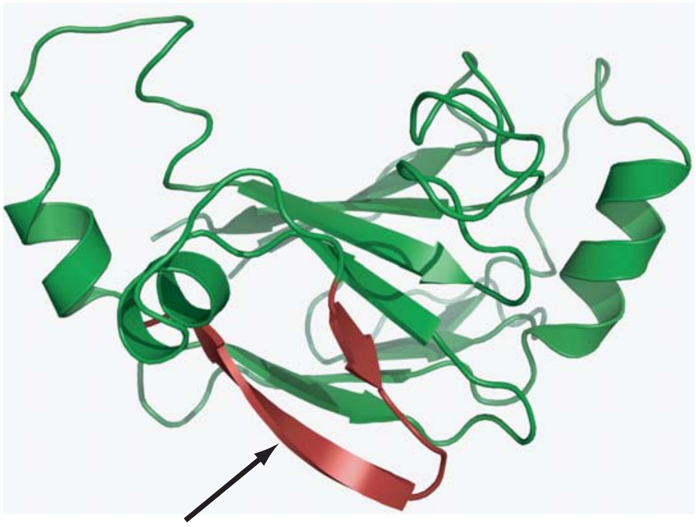

Supplementary Fig. S2. **Amino acids 400 to 441 of Smad6 show a β-sheet structure.** Homology modeling of the Smad6 MH2 domain. The arrow indicates amino acids 400 to 441 of the Smad6 MH2 domain with a β-sheet structure.

Supplementary Fig.S3

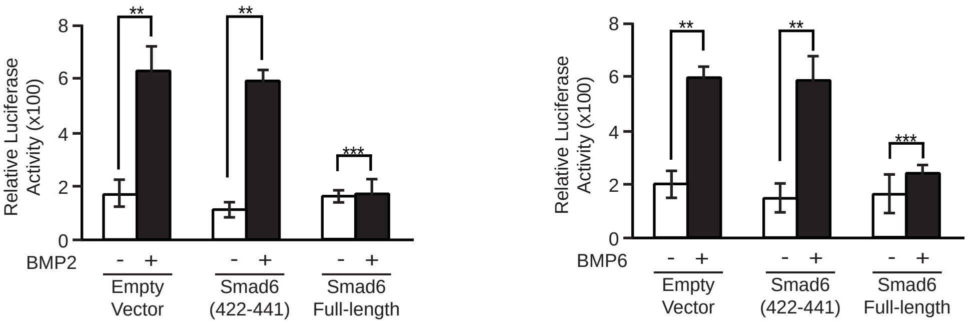

Supplementary Fig. S3. The minimal region of Smad6 (422-441) does not inhibit BMP signaling. The BRE-Luc reporter plasmid was co-transfected with an empty vector or the Myc-Smad6(422-441) plasmid or full-length Smad6 into RAW264.7 cells, respectively. After 24 h, cells were treated with BMP2 or BMP6 for 6 h and luciferase activity was measured and normalized. The data were statistically analyzed by a *t*-test and show the mean ±S.D. of three independent experiments. ***P*<0.005, **P*<0.05 compared to the control (no treatment of BMP2 or BMP6).

A

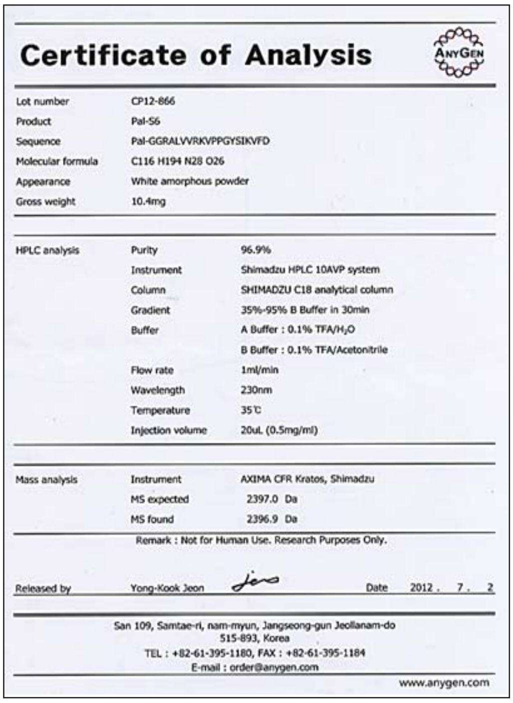

Certificate of Analysis

Lot number	CP12-866	
Product	Pal-S6	
Sequence	Pal-GGRALVVRKVPPGYSIKVFD	
Molecular formula	C116 H194 N28 O26	
Appearance	White amorphous powder	
Gross weight	10.4mg	
HPLC analysis	Purity	96.9%
	Instrument	Shimadzu HPLC 10AVP system
	Column	SHIMADZU C18 analytical column
	Gradient	35%-95% B Buffer in 30min
	Buffer	A Buffer : 0.1% TFA/H_2O
		B Buffer : 0.1% TFA/Acetonitrile
	Flow rate	1ml/min
	Wavelength	230nm
	Temperature	35℃
	Injection volume	20uL (0.5mg/ml)
Mass analysis	Instrument	AXIMA CFR Kratos, Shimadzu
	MS expected	2397.0 Da
	MS found	2396.9 Da

Remark : Not for Human Use. Research Purposes Only.

Released by Yong-Kook Jeon Date 2012 . 7 . 2

San 109, Samtae-ri, nam-myun, Jangseong-gun Jeollanam-do
515-893, Korea
TEL : +82-61-395-1180, FAX : +82-61-395-1184
E-mail : order@anygen.com

www.anygen.com

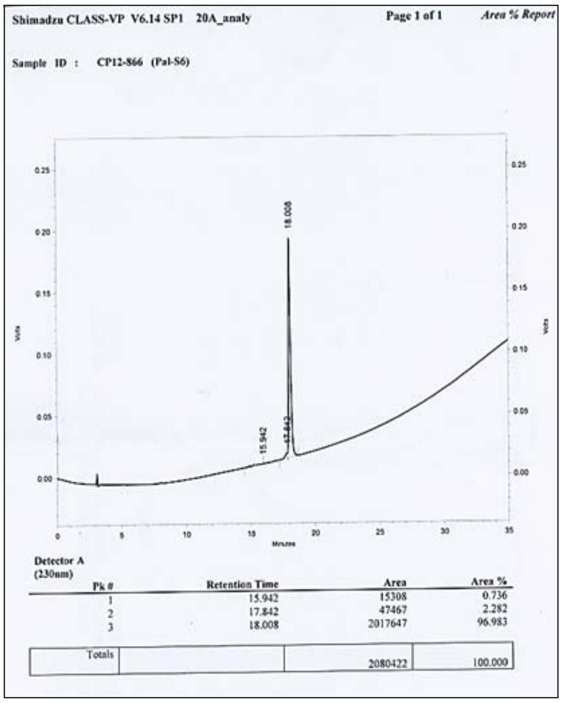

Pk #	Retention Time	Area	Area %
1	15.942	15308	0.736
2	17.842	47467	2.282
3	18.008	2017647	96.983
Totals		2080422	100.000

B

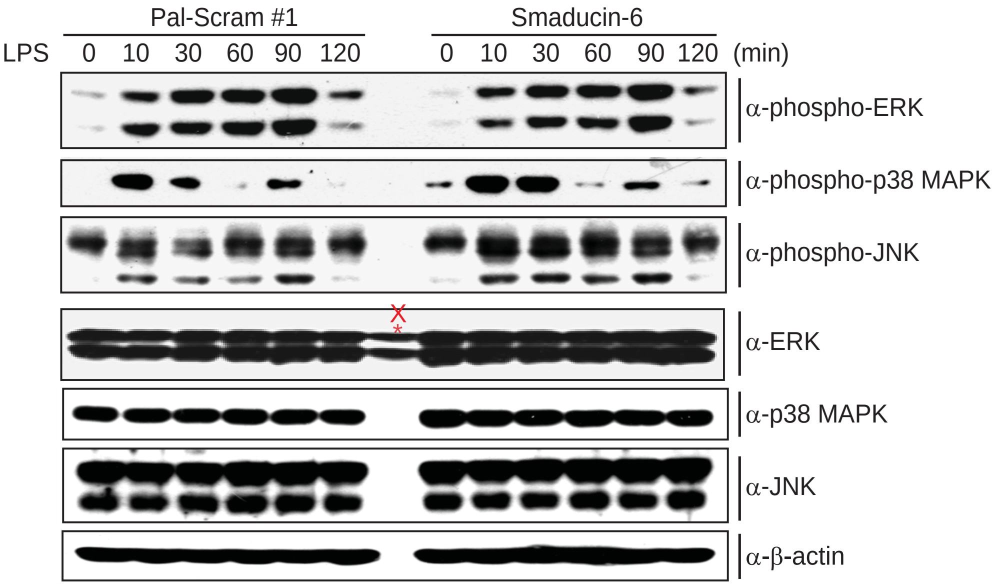

X * :Thiese bands were leakages of adjacent wells and irrelevant to the experiment.

Supplementary Fig. S4. Smaducin-6 is not involved in the regulation of LPS-induced activation of MAP kinases.

A. Data sheet showing the quality of the synthesized Smaducin-6 peptide. Smaducin-6 at a purity of over 95 % was used in this study.

B. RAW264.7 cells were pre-treated with 100 nM Pal-Scram #1 peptide or Smaducin-6, and subsequently treated with LPS for the indicated time. Cell lysates were immunoblotted with the indicated antibodies against endogenous proteins. β-actin was used as a loading control. The data are representative of three independent experiments.

Supplementary Fig. S5

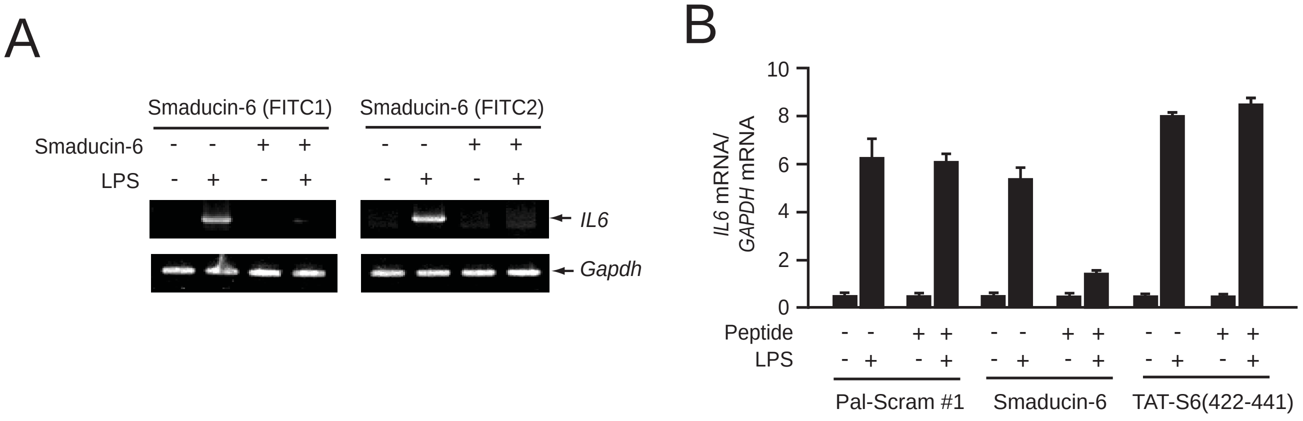

Supplementary Fig. S5. A TAT-conjugated peptide, composed of Smad6 amino acids 422 to 441, does not reduce LPS-induced *IL6* gene expression.
A. FITC-conjugated Smaducin-6 decreased LPS-induced *IL6* gene expression.
B. A TAT-conjugated peptide, composed of Smad6 amino acids 422 to 441 [TAT-S6(422-441)], did not inhibit LPS-induced *IL6* gene expression. After RAW264.7 cells were pretreated with 100 nM FITC-conjugated Smaducin-6 or TAT-S6(422-441) peptide for 30 min, cells were treated with LPS for 2 h. *IL-6* expression was analyzed by (**A**) RT-PCR and (**B**) quantitative real time RT-PCR. Expression of the *GAPDH* gene was used as a loading control. The data in (**A**) are representative of three independent experiments. The data in (**B**) show the mean ±S.D. of three independent experiments.

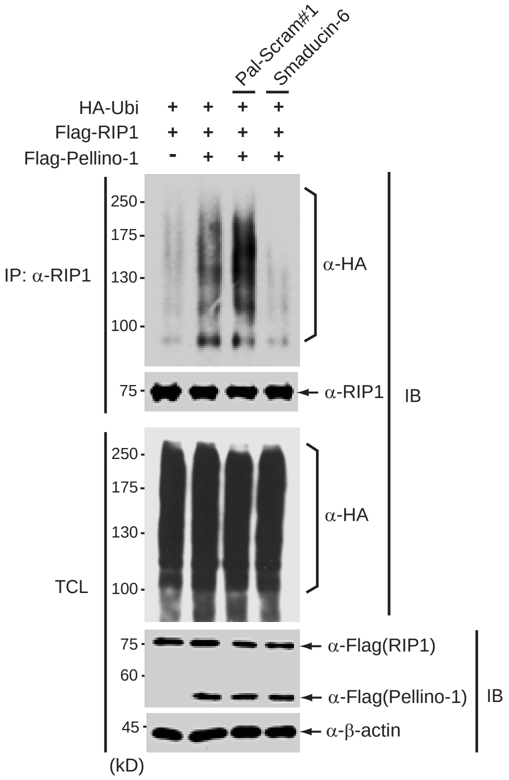

Supplementary Fig. S6. Smaducin-6 decreases the polyubiquitination of RIP1.
HEK293 cells were pre-treated with 100 nM scrambled peptide (Pal-Scram #1) or Smaducin-6 for 30 min, and plasmids encoding HA-tagged ubiquitin (Ubi), Flag-RIP1 and Flag-Pellino-1 were transiently transfected. After 24 h, cells were harvested to examine the polyubiquitination of RIP1 protein. Cell lysates were immunoprecipitated (IP) with anti-RIP1 antibody and immunoblotted (IB) with the indicated antibodies. Total cell lysates (TCL) were immunoblotted by the indicated antibodies. Data are representative of at least three independent experiments.

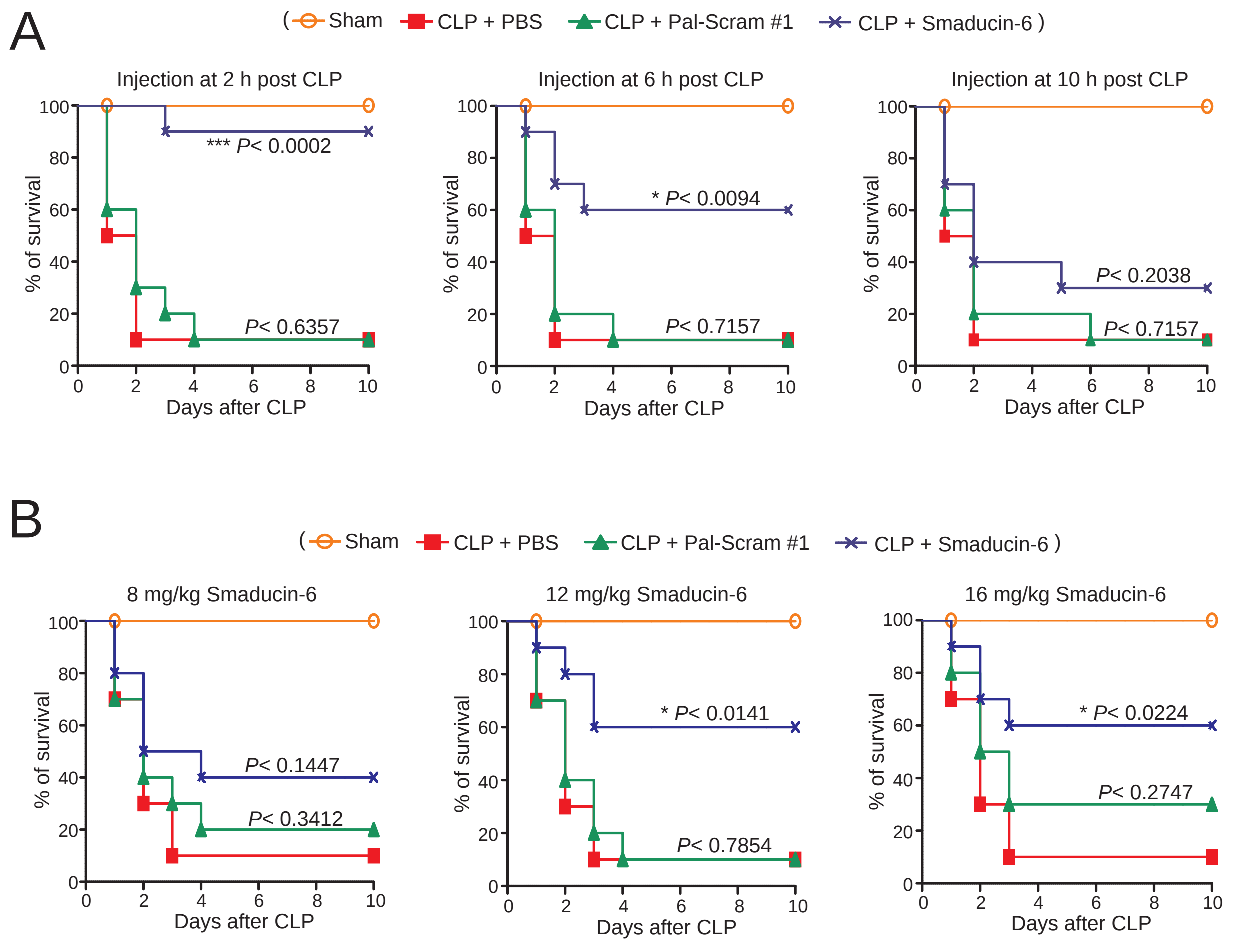

Supplementary Fig. S7. Determination of effective injection time of Smaducin-6 and therapeutic effects of Smaducin-6 intravenously injected.

A. Determination of effective injection time post-CLP. Smaducin-6 (100 μg) was subcutaneously injected at 2 h, 6 h, and 10 h after severe CLP in BALB/c mice. After the initial injection, Smaducin-6 was subcutaneously injected three times at 12 h intervals. The total amount of Smaducin-6 injected into CLP mice was 16 mg/kg. n=10 mice per group per experiment. **B.** Intravenous injections of Smaducin-6 peptide slightly increase the survival rate of severe CLP-induced sepsis mice (BALB/c). Smaducin-6 peptide was intravenously injected via the tail vein at 2 h post-CLP and repeatedly injected three times at 12 h intervals. The noted concentrations indicate total amounts injected. n=10 mice per group per experiment. Data in (**A**) and (**B**) were statistically analyzed by the log-rank test. ***P<0.001, **P<0.005, *P<0.05 compared to vehicle control (CLP+Pal-Scram #1).

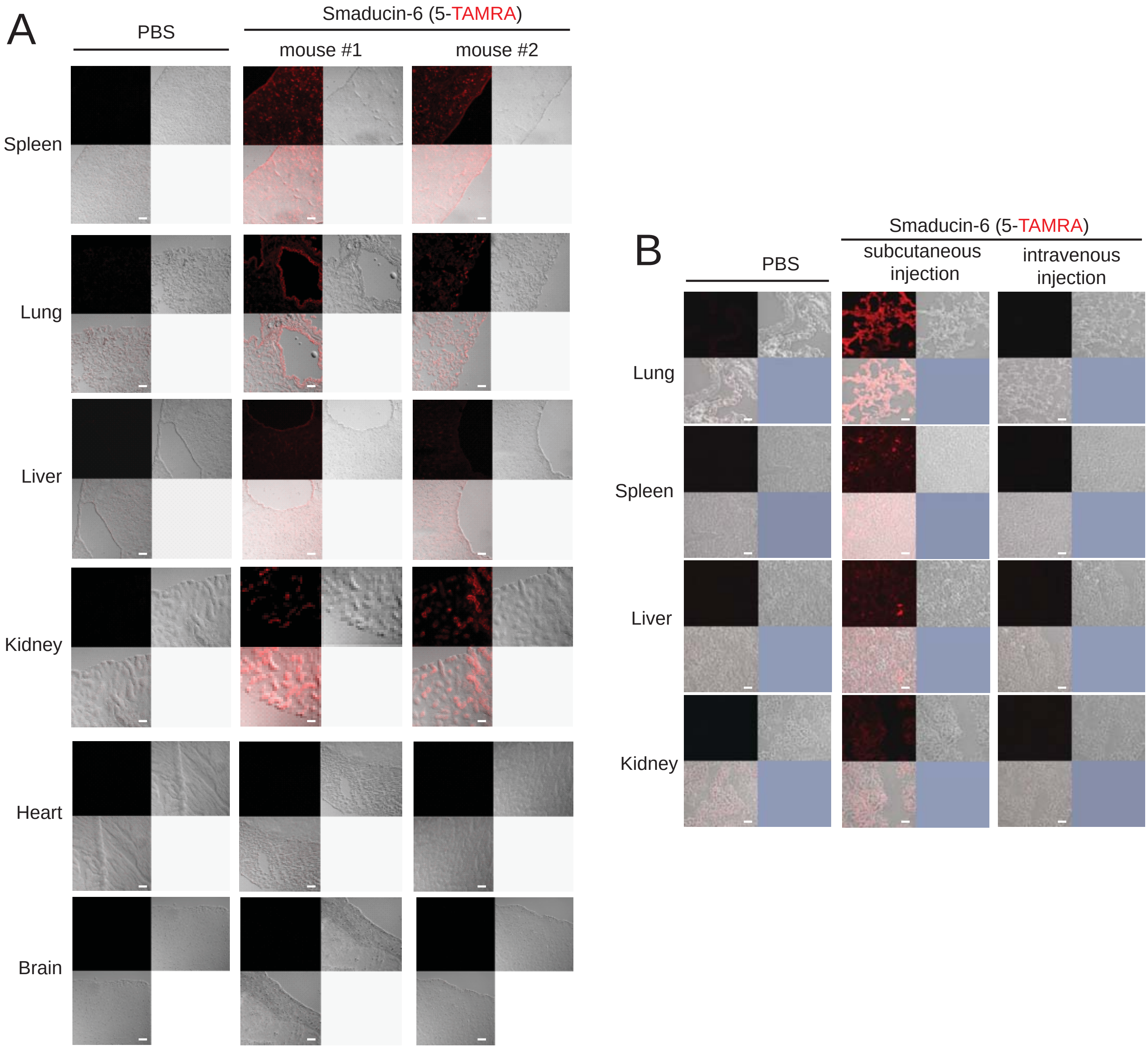

Supplementary Fig. S8. Biodistribution of Smaducin-6 upon subcutaneous injection or intravenous injection.
A. One hour after subcutaneous injection of 5-TAMRA-conjugated Smaducin-6 into normal BALB/c mice, biodistribution of the Smaducin-6 peptide was observed in the indicated tissues by confocal microscopy. Magnification, x200. Data are representative of three independent experiments.
B. One hour after subcutaneous or intravenous injection of 5-TAMRA-conjugated Smaducin-6 into normal BALB/c mice, biodistribution of the Smaducin-6 peptide was observed in the lung, spleen, liver, and kidney by confocal microscopy. Injection of PBS buffer was used as a control. Scale bar; 100 μm (Magnification; x200). Data are representative of three independent experiments.

Supplementary Fig. S9

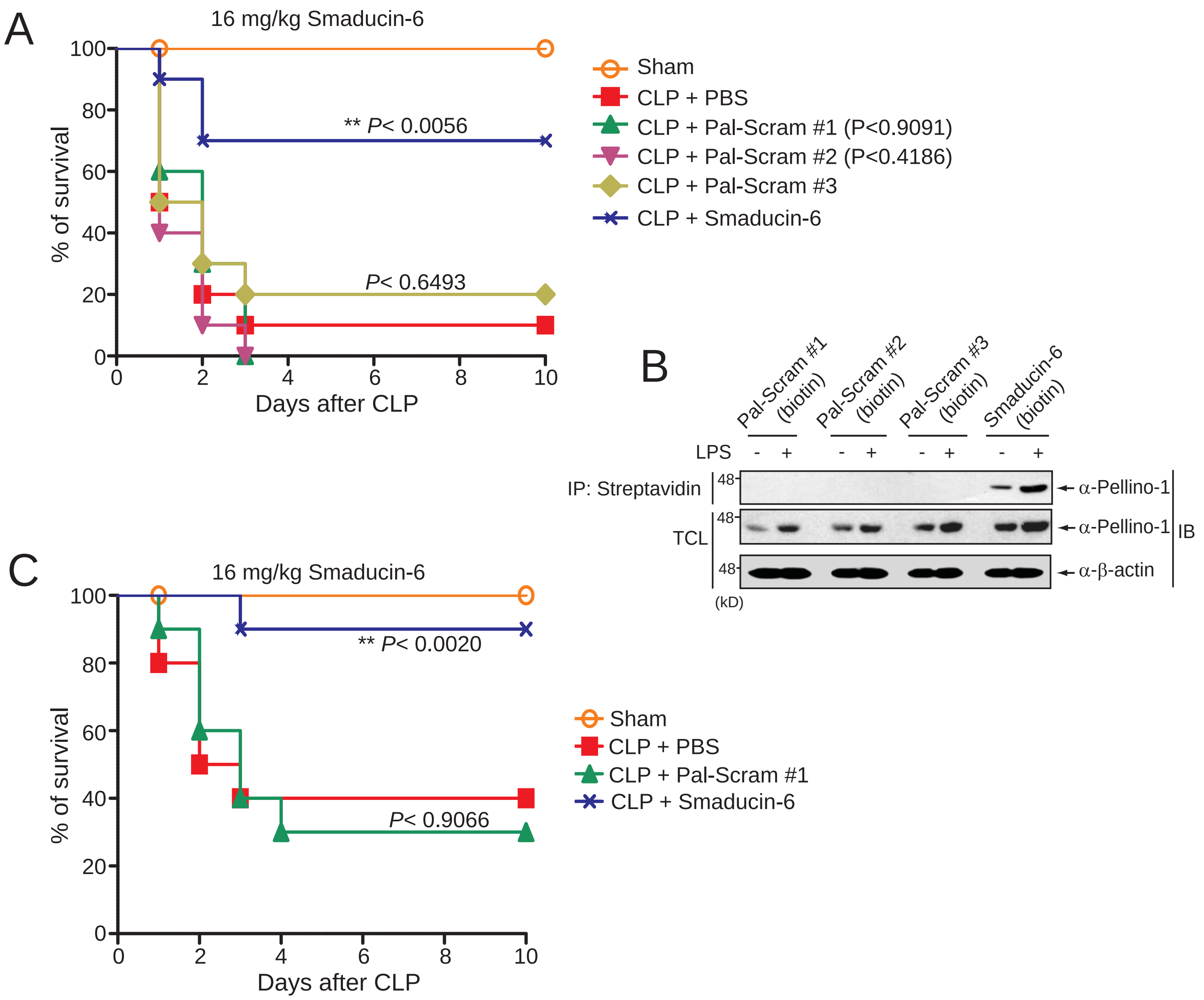

Supplementary Fig. S9. Smaducin-6 protects mice from CLP-induced sepsis through binding to Pellino-1.
A. Subcutaneous injections of different scrambled peptides do not show protective effects in CLP-induced sepsis mice (BALB/c mice). Each scrambled peptide (100 μg) or Smaducin-6 was initially injected at 2 h post-CLP. The injection was repeated three times at 12 h intervals, resulting in the injection of total 16 mg/kg scrambled peptide. n=10 mice per group per experiment. Data were statistically analyzed by the log-rank test. ***P*<0.005 compared to each vehicle control (CLP+Pal-Scram peptide). **B.** After pre-treating RAW264.7 cells with biotin-conjugated scrambled peptides (100 nM Pal-Scram #1, #2, #3) or Smaducin-6 (100 nM) for 30 min, cells were treated with LPS for 2 h. Subsequent precipitation by streptavidin-agarose showed that endogenous Pellino-1 binds to the Smaducin-6 peptide. **C.** Subcutaneous injection of Smaducin-6 (total 16 mg/kg) increases the survival rate of less severe CLP-induced sepsis mice (BALB/c mice). n=10 mice per group per experiment. Data were statistically analyzed by the log-rank test. ***P*<0.005 compared to vehicle control (CLP+Pal-Scram #1).

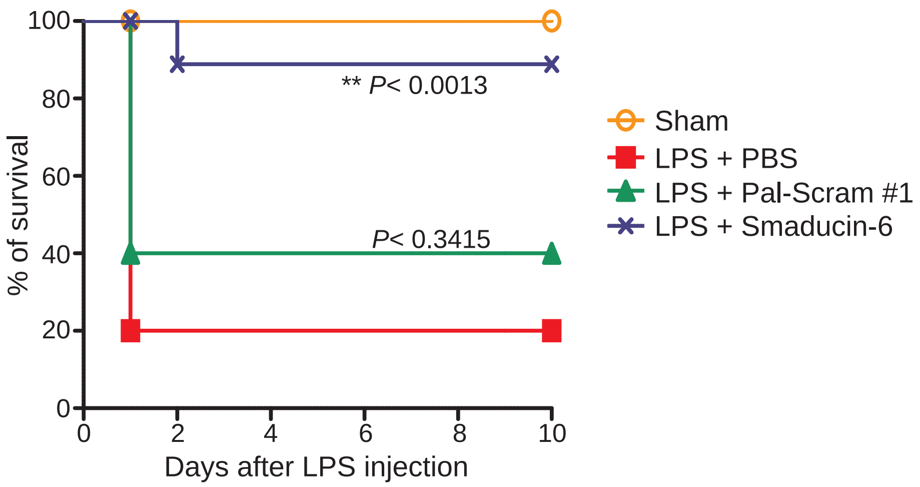

Supplementary Fig. S10. Smaducin-6 treatment increases the survival rate of LPS-induced septic shock mice.
For an endotoxic septic shock model, 60 mg/kg of LPS was intraperitoneally injected into BALB/c mice (Kim *et al,* 2010). After 2 h, 100 μg of Smaducin-6 was injected subcutaneously four times at 12 h intervals (total 16 mg/kg). The survival rate was monitored daily for 10 days. A scrambled peptide (Pal-Scram #1) was used as a negative control. n=10 mice per group per experiment. The data were statistically analyzed by the log-rank test. **P<0.005 compared to vehicle control (LSP+Pal-Scram #1).

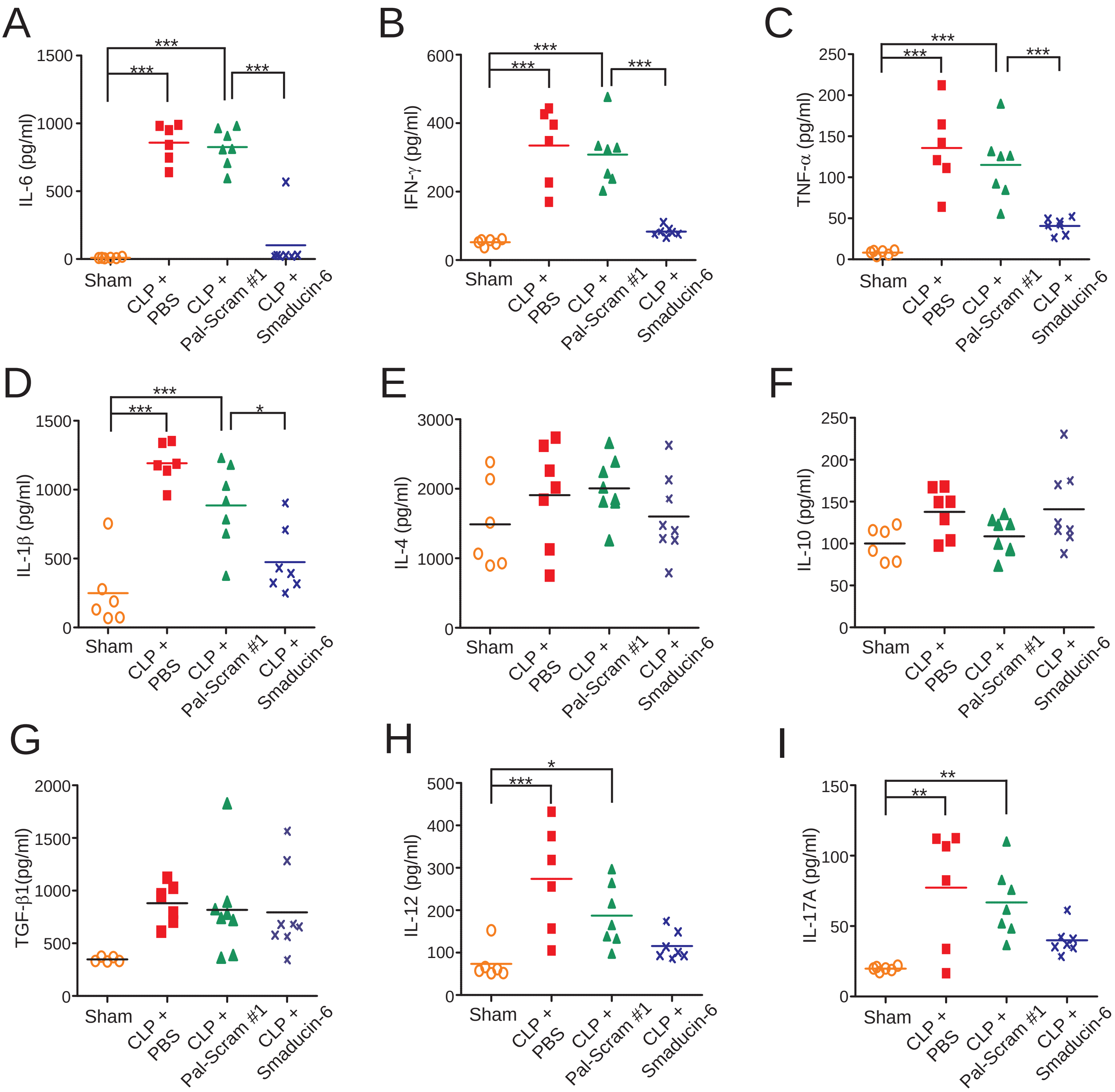

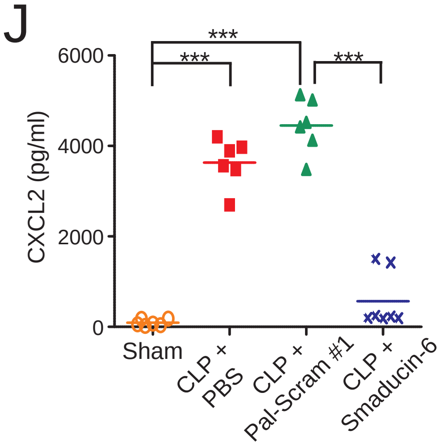

Supplementary Fig. S11. Smaducin-6 reduces pro-inflammatory cytokines in the peritoneal lavage fluid of CLP mice.
A-J. Smaducin-6 or Pal-Scram #1 peptide was subcutaneously injected two times at 2 h and 14 h post-CLP. Peritoneal lavage fluid was collected for ELISA analysis of each cytokine 24 h post-CLP. n=10 mice per group per experiment. The data were statistically analyzed by the Dunnetts Multiple Comparison test (1-way ANOVA). ***P<0.001, **P<0.05, *P<0.5. (**A**) IL-6 (**B**) IFN-γ (**C**) TNF-α (**D**) IL-1β (**E**) IL-4 (**F**) IL-10 (**G**) TGF-β (**H**) IL-12 (**I**) IL-17A (**J**) CXCL2.

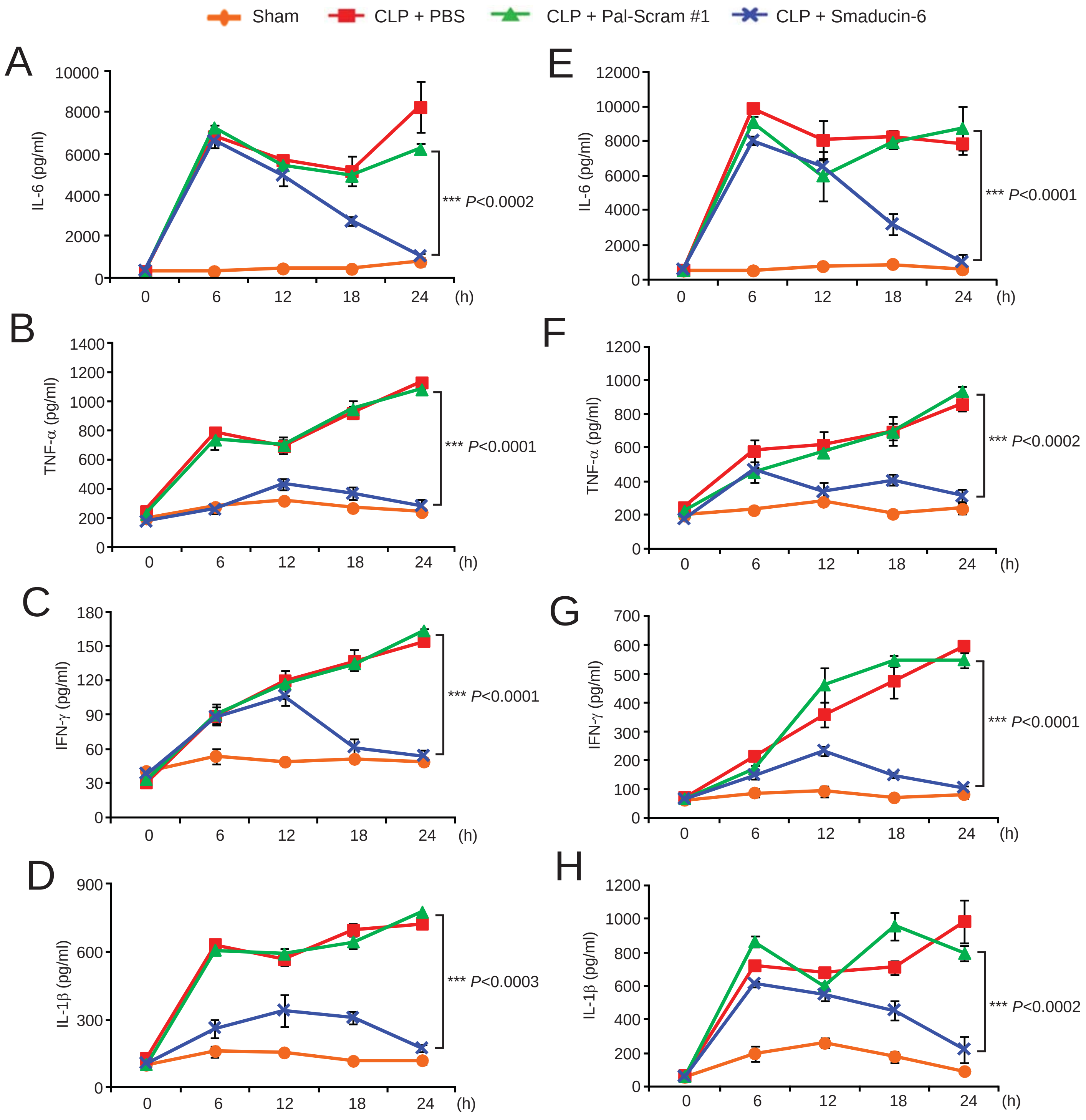

Supplementary Fig. S12. Time-dependent inhibition of pro-inflammatory cytokines by Smaducin-6 in CLP mice.
Smaducin-6 or Pal-Scram #1 peptide was subcutaneously injected two times at 2 h and 14 h post-CLP. Blood (**A**-**D**) or peritoneal lavage fluid (**E**-**H**) was collected for ELISA analysis of each pro-inflammatory cytokine at 6 h, 12 h, 18 h, and 24 h post-CLP. n=5 mice per group per experiment. Data were statistically analyzed by the log-rank test. The data show the mean ±S.D. of these experiments. ****P*<0.001 compared to vehicle control (CLP+Pal-Scram #1).

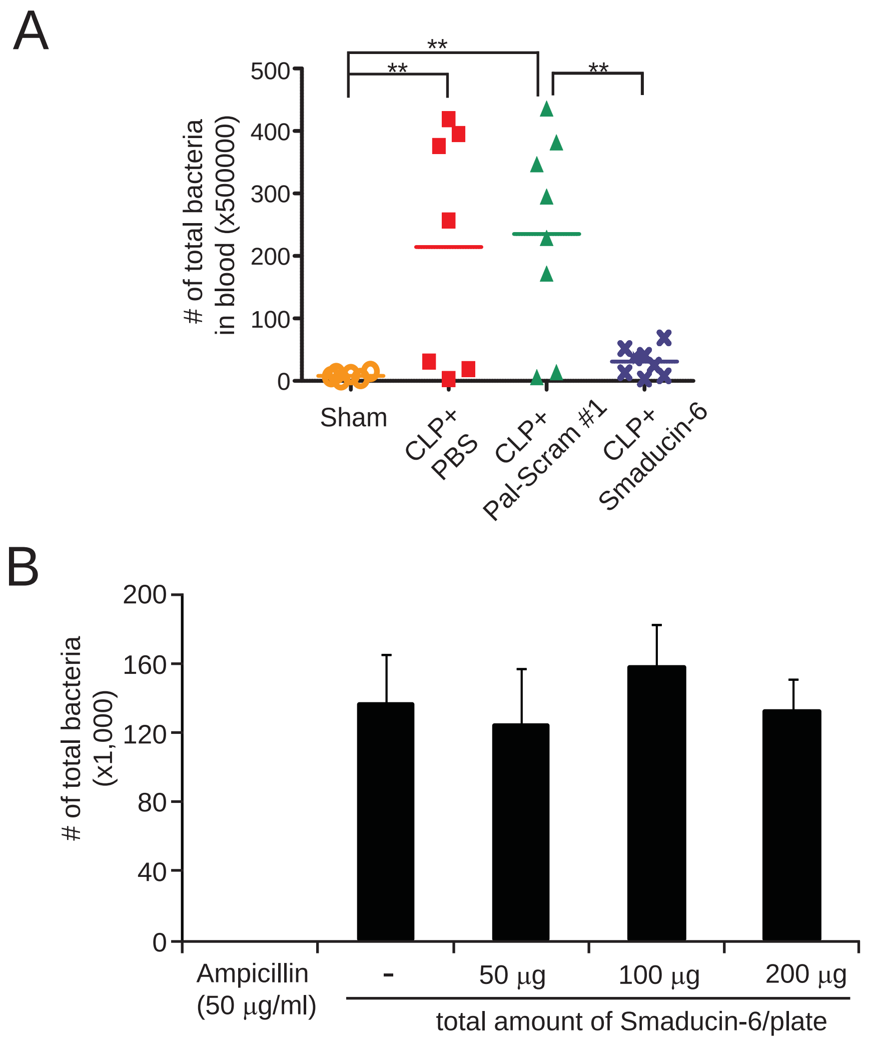

Supplementary Fig. S13. Smaducin-6 reduces bacterial loads in the blood of CLP mice.
A. Bacterial loads in the blood of CLP mice treated with Pal-Scram #1 or Smaducin-6 peptide were analyzed by colony counting. n=10 mice per group per experiment. Data was statistically analyzed by the Mann-Whitney *U* test. ***P*<0.005 compared to sham or vehicle control (CLP+Pal-Scram #1).
B. A direct killing effect of Smaducin-6 was examined against gram-negative bacteria *E. coli* DH5α. The indicated amounts of Smaducin-6 were loaded together with *E. coli* DH5α on Luria-Bertani (LB) plates and the surviving colonies were counted. As a control, an LB plate containing antibiotics (ampicillin) was used. Data show the mean ±S.D. of three independent experiments.

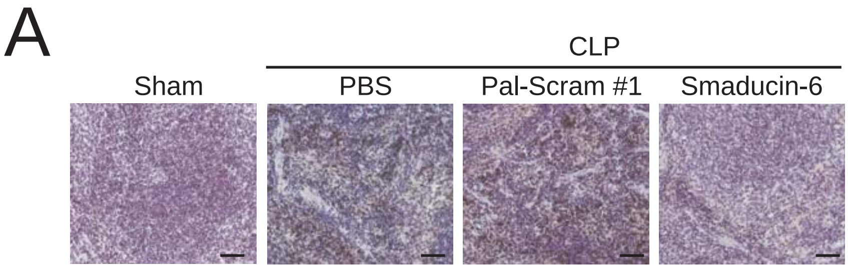

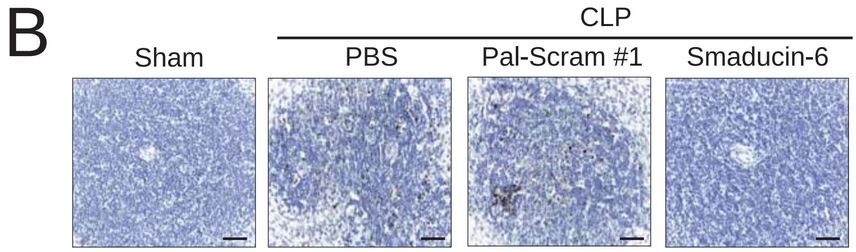

Supplementary Fig. S14. Smaducin-6 reduces the levels of apoptosis induced by sepsis.
A. TUNEL assays in the spleens of CLP mice treated with Smaducin-6, Pal-Scram #1, or PBS.
B. Immunohistochemical (IHC) analysis of the spleens of CLP mice treated with Smaducin-6, Pal-Scram #1, or PBS. Scale bar, 100 μm (Magnification, x200). Data are representative of three independent experiments.

Supplementary Fig. S15

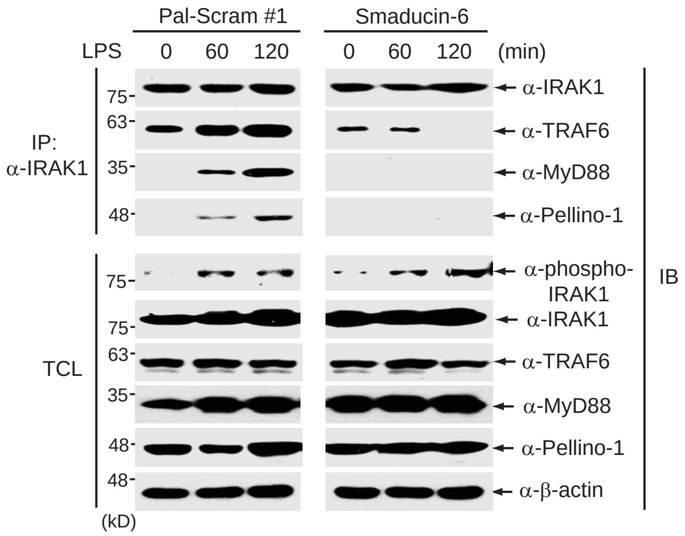

Supplementary Fig. S15. Smaducin-6 does not affect IRAK1 phosphorylation upon LPS treatment. After pre-treating RAW264.7 cells with 100 nM Pal-Scram #1 or Smaducin-6 for 30 min, cells were treated with LPS for the indicated time. Cell lysates were immunoprecipitated (IP) with anti-IRAK1 antibody against endogenous proteins and subsequently immunoblotted (IB) with the indicated antibodies. Total cell lysates (TCL) were also immunoblotted with the indicated antibodies. Although Smaducin-6 disrupted IRAK1-mediated signaling complexes, it did not change IRAK1 phosphorylation levels. Data are representative of three independent experiments.

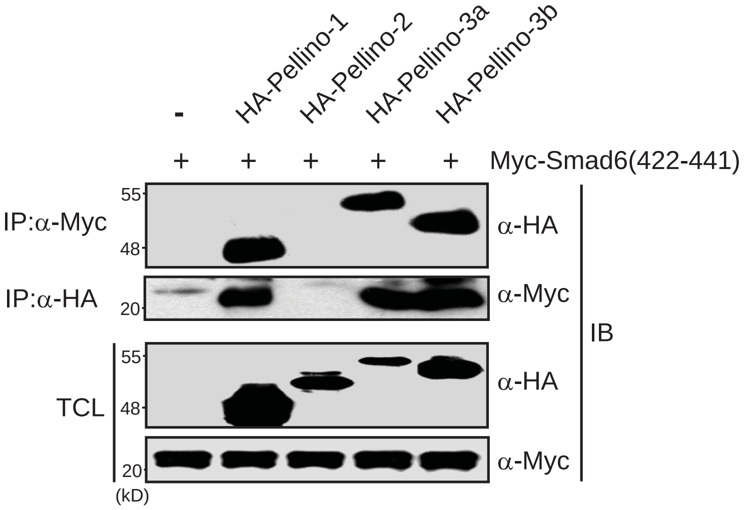

Supplementary Fig. S16. The minimal region of Smad6 (amino acids 422 to 441), which composes Smaducin-6, binds to Pellino-3, but not Pellino-2.
A plasmid encoding the minimal region of Smad6 (amino acids 442 to 441) was co-transfected into HEK293 cells with HA-Pellino-1, HA-Pellino-2, HA-Pellino-3a, and HA-Pellno-3b, respectively. Cell lysates were immunoprecipitated with anti-Myc antibody and immunoblotted with anti-HA antibody, or immunoprecipitated with anti-HA antibody and immunoblotted with anti-Myc antibody. Data are representative of at least three independent experiments. IP, immunoprecipitation; IB, immunoblot; TCL, total cell lysates.

Supplementary Table S1. Peptide sequences used in this study

Peptides	Amino acid Sequence (N terminal to C-terminal)
Smaducin-6	Palmitic acid-GGRALVVRKVPPGYSIKVFD
Smaducin-6 (FITC-1*)	Palmitic acid-GGRALVVRK*VPPGYSIKVFD
Smaducin-6 (FITC-2*)	Palmitic acid-GGRALVVRKVPPGYSIK*VFD
Smaducin-6 (TAMRA*)	Palmitic acid-GGRALVVRK*VPPGYSIKVFD
Smaducin-6 (Biotin*)	Palmitic acid-GGRALVVRK*VPPGYSIKVFD
TAT-S6(422-441)	GRKKRRQRRRPQ-GGRALVVRKVPPGYSIKVFD
TAT-S6(422-441) (FITC*)	GRKKRRQRRRPQ-GGRALVVRK*VPPGYSIKVFD
Pal-Scram #1	Palmitic acid-VDKFRVVKVILSAYRGGPGP
Pal-Scram #2	Palmitic acid-VGKRGDFVYKIVGPVLARSP
Pal-Scram #3	Palmitic acid-VPSGKRARGLVDFPVYVIGK
Pal-Scram #1 (Biotin*)	Palmitic acid-VDKFRVVK*VILSAYRGGPGP
Pal-Scram #2 (Biotin*)	Palmitic acid-VGK*RGDFVYKIVGPVLARSP
Pal-Scram #3 (Biotin*)	Palmitic acid-VPSGK*RARGLVDFPVYVIGK

(* indicate the lysine residue which is conjugated with the corresponding marker.)

Supplementary Table S2. Primers used in this study

Constructs	Direction	Sequence (5′ - 3′)
HA-Pellino-1	Forward	ATGTTTTCTCCTGATCAA
	Reverse	TTAGTCTAGAGGTCCTTG
HA-Pellino-1 N	Forward	ATGTTTTCTCCTGATCAA
	Reverse	TCTGCAGGCAAATCTTGA
Myc-Smad6 MH2	Forward	TGGTGCAGCGTGGCGTA
	Reverse	CTATCTGTGGTTGTTGAGTA
Myc-Smad6 346F	Forward	CGCCTCTATGCGGTGTAC
	Reverse	CTATCTGTGGTTGTTGAGTA
Myc-Smad6 371F	Forward	CAGCTCAACCTGGAGCAG
	Reverse	CTATCTGTGGTTGTTGAGTA
Myc-Smad6 385F	Forward	CGCAGCAAGATCGGTTTT
	Reverse	CTATCTGTGGTTGTTGAGTA
Myc-Smad6 464R	Forward	TGGTGCAGCGTGGCGTA
	Reverse	GCGCACACTGTGCGGGTC
Myc-Smad6 441R	Forward	TGGTGCAGCGTGGCGTA
	Reverse	GTCGAACACCTTGATGGA
Myc-Smad6 410R	Forward	TGGTGCAGCGTGGCGTA
	Reverse	GGGGTGCTCGCCCCGGTT
Myc-Smad6 385-441	Forward	CGCAGCAAGATCGGTTTT
	Reverse	GTCGAACACCTTGATGGA
Myc-Smad6 385-427	Forward	CGCAGCAAGATCGGTTTT
	Reverse	GACCAGGGCGCGGCCTCC
Myc-Smad6 385-418	Forward	CGCAGCAAGATCGGTTTT
	Reverse	CAGCGTCGGGGAGTTGAC
Myc-Smad6 385-410	Forward	CGCAGCAAGATCGGTTTT
	Reverse	GGGGTGCTCGCCCCGGTT
Myc-Smad6 400-441	Forward	GGCGTGTGGGCCTACAAC
	Reverse	GTCGAACACCTTGATGGA
Myc-Smad6 400-427	Forward	GGCGTGTGGGCCTACAAC
	Reverse	GACCAGGGCGCGGCCTCC
Myc-Smad6	Forward	GGCGTGTGGGCCTACAAC

400-418	Reverse	CAGCGTCGGGGAGTTGAC
Myc-Smad6 400-410	Forward	GGCGTGTGGGCCTACAAC
	Reverse	GGGGTGCTCGCCCCGGTT
Myc-Smad6 422-441	Forward	GCGATCGCGGGCAGGCGC
	Reverse	GTCGAACACCTTGATGGA
HA-Pellino-2	Forward	ATGTTTTCCCCTGGCCAG
	Reverse	TCAGTCAATTGGACCTTGG
HA-Pellino-3a	Forward	ATGGTGCTGGAAGGAAACCC
	Reverse	CTAATCCAGCGGGCCGG
HA-Pellino-3b	Forward	ATGGTGCTGGAAGGAAACCC
	Reverse	CTAATCCAGCGGGCCGG

Supplementary Materials and methods

Bacteria counts

Peritoneal lavage fluids and blood were collected 24 h after CLP. Samples were separated by centrifugation at 13,000 rpm for 10 min. Diluted samples were cultured on agar base dishes and incubated at 37 °C for 24 h. Bacterial counts were determined by counting colony-forming units.

Flow cytometry analysis

Ten thousand human neutrophils and mouse neutrophils were stained with PE-conjugated anti-human CXCR2 antibody (FAB331P, R&D Systems, 10 μl/10^6 cells) and PE-conjugated anti-mouse CXCR2 antibody (FAB2164P, R&D Systems, 10 μl/10^6 cells), respectively. Mouse splenocytes were stained with primary rabbit anti-TRAIL antibody (C92B9, Cell Signaling, dilution ratio 1:200) and secondary FITC-conjugated goat anti-mouse IgG antibody (NOVUS, 1:500), and FITC-conjugated anti-mouse IFN-β antibody (22400-3, Interferon Source, 1:200). Cells were analyzed by the FACScalibur flow cytometer and CellQuest Pro software (BD Bioscience) was used for data analysis.

Immunohistochemistry

Paraffin-embedded slides were deparaffinized. Antigen unmasking was carried out by microwaving in 10 mM sodium citrate buffer. Slides were incubated with primary antibodies in PBS containing 5 % normal goat serum at 4 °C overnight and subsequent procedures followed the protocol of the Vectastain ABC kit (Vector Lab). Slides were counterstained with hematoxylin. Antibodies for cleaved Caspase-3 (#9661, Cell Signaling, dilution ratio 1:300) and TRAIL (AF1121, R&D systems, 1:200) were used.

Immunofluorescence

After subcutaneous injection of 100 μg/kg 5-TAMRA-conjugated Smaducin-6 peptide, several tissues (spleen, lung, liver, kidney, heart and brain) were isolated and molded with OCT-compound and fast frozen in dry ice. Tissues were cut and mounted on a slide glass and observed by confocal microscopy (Carl Zeiss).

Modeling of Smad6 MH2 domain

Homology modeling of the Smad6 domain was performed on the SWISS-MODEL Workspace server (Arnold *et al*, 2006).

Transient transfection and reporter assay

All plasmids were transiently transfected into HEK293, CMT-93 cells or RAW264.7 cells using Effectene (Qiagen). Cells were treated for 2 h with LPS (10 ng/ml) or TGF-β1 (5 ng/ml). For certain experiments, cells were pre-treated with 100 nM Pal-Scram peptide or Smaducin-6 peptide for 30 min before 10 ng/ml LPS treatment. Luciferase activity was normalized to β-galactosidase activity to adjust for variations in transfection efficiency. All experiments were independently repeated at least three times with similar results.

Peli1 knockdown, RNA extraction and quantitative real-time RT-PCR

Small interfering RNAs (siRNAs) used to silence human *Peli1* gene in human monocyte THP1 cells were obtained commercially (Catalog No. SI04284665, Qiagen). Before siRNA transfection, THP1 cells were differentiated with 100 nM phobol 12 -myristate 13-acetate (PMA) overnight and siRNAs were transfected with Lipofectamine RNAiMAX (Invitrogen) for 24 h. After washing with PBS, cells were incubated for 2 additional days. At this time,

THP1 cells were pre-treated with 100 nM Smaducin-6 and Pal-Scram peptide for 30 min, and subsequently treated with LPS for 2 h. siRNAs used as the negative control (si-CON) were provided by Qiagen. Total RNA was isolated using the TRIZOL reagent (Invitrogen). The Superscript kit (Invitrogen) was used for reverse transcription. Real-time RT-PCR experiments were performed essentially as described (Choi *et al*, 2006). Primer sequences of the *IL6* and *GAPDH* genes were previously described (Lee *et al*, 2011). Primer sequences of human *Peli1* gene were as follows: Forward; 5'-ATCTTTGTTCACTGGTCAGGAG-3', Reverse; 5'-CACAACACTTCACCCTCTCG-3'. For quantitative RT-PCR, an iCycler real-time PCR machine and iQ SYBR Green Supermix (Bio-Rad) were used to measure the expression of genes under the following conditions; 45 cycles of 95 °C for 30 s, 62 °C for 30 s, and 72 °C for 30 s. All reactions were independently repeated at least three times to ensure reproducibility.
