# Supplementary figures and images for: Inhibition of lethal inflammatory responses through the targeting of membrane-associated Toll-like receptor 4 signaling complexes with a Smad6-derived peptide
Source: EMBO Mol Med. 2015 Mar 12;7(5):577–92. doi: 10.15252/emmm.201404653 (PMC4492818; doi:10.15252/emmm.201404653)

**A**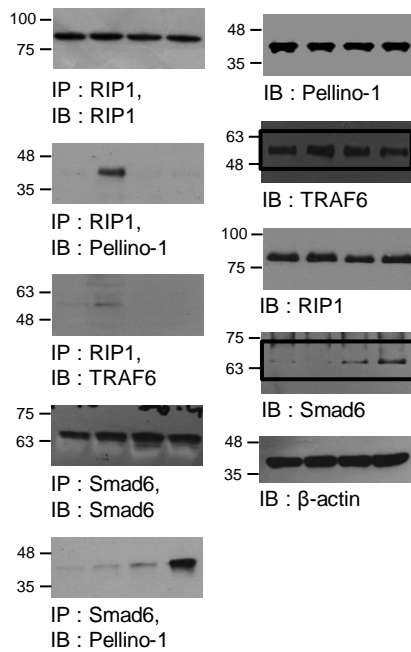**B**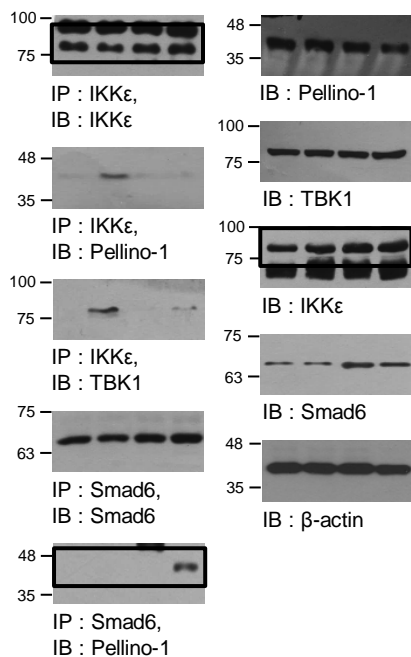**C**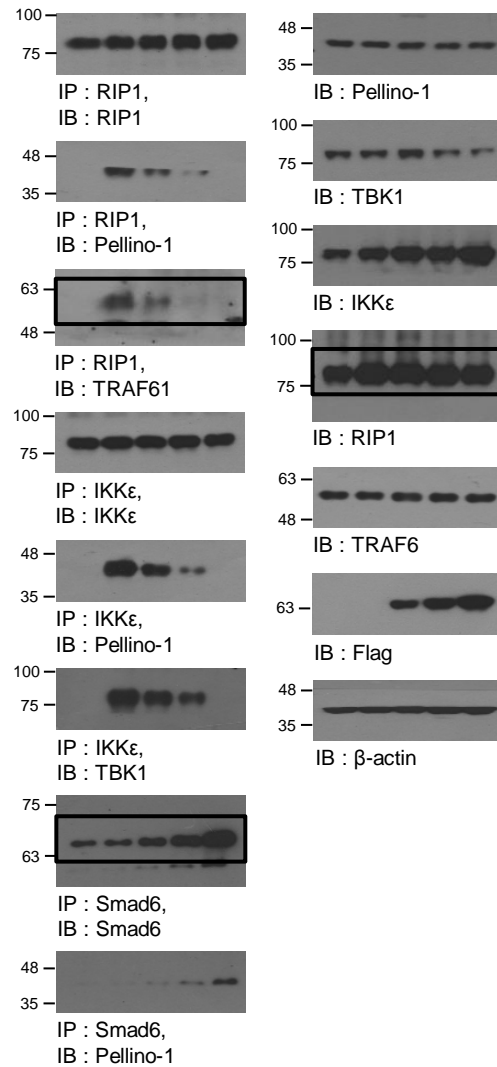

Supplement: Supplementary file 2 [file emmm0007-0577-sd2.pdf]

B

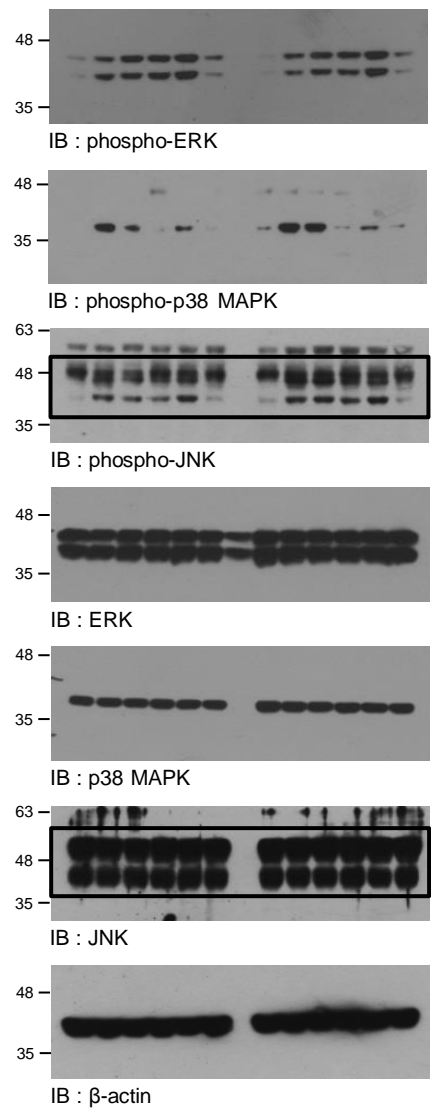

Supplement: Supplementary file 3 [file emmm0007-0577-sd3.pdf]

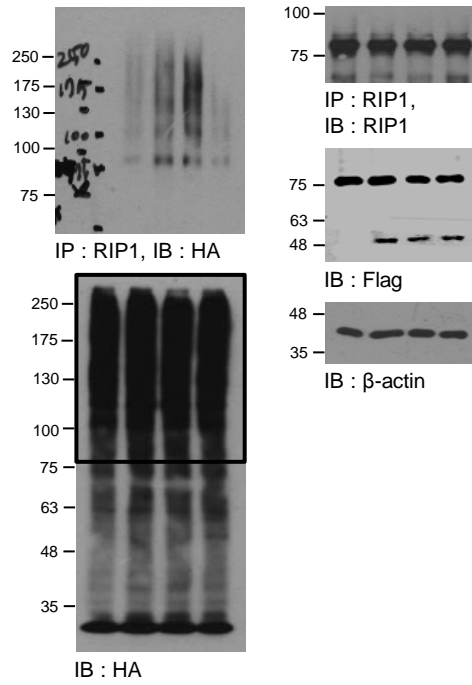

Supplement: Supplementary file 4 [file emmm0007-0577-sd4.pdf]

**B**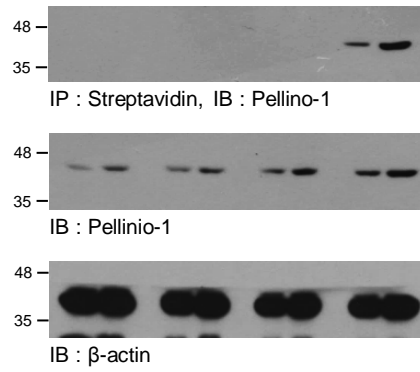

Supplement: Supplementary file 5 [file emmm0007-0577-sd5.pdf]

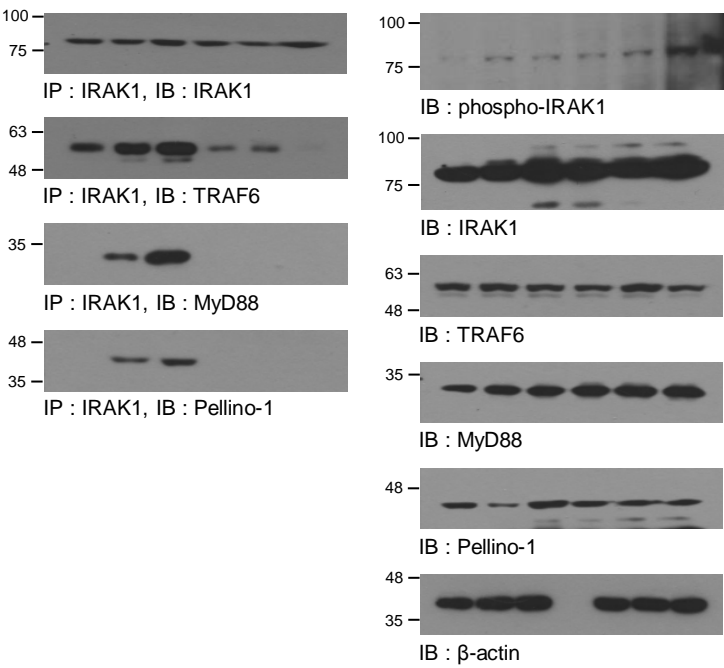

Supplement: Supplementary file 6 [file emmm0007-0577-sd6.pdf]

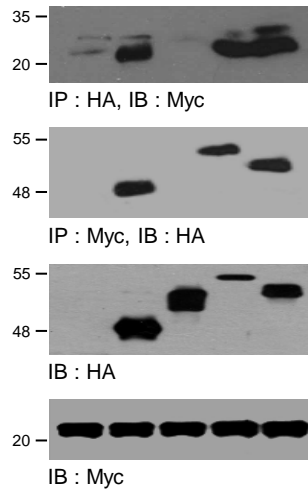

Supplement: Supplementary file 7 [file emmm0007-0577-sd7.pdf]

**B**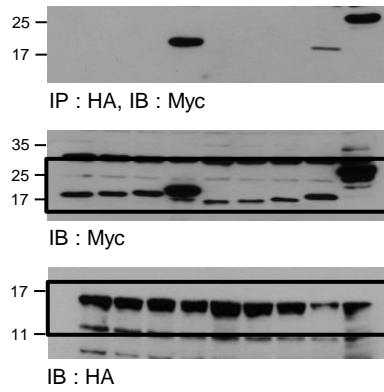**E**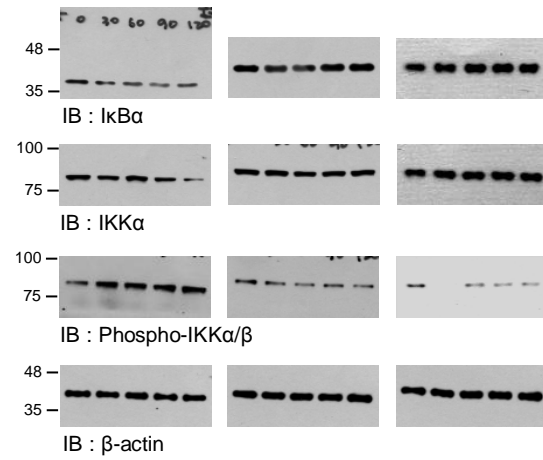

Supplement: Supplementary file 9 [file emmm0007-0577-sd9.pdf]

**B**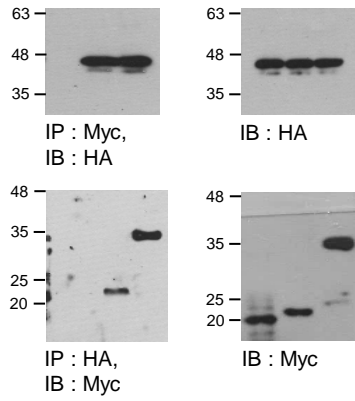**C**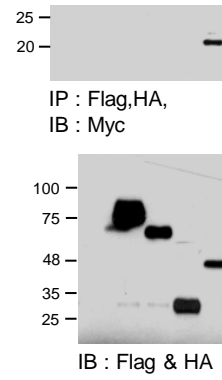**G**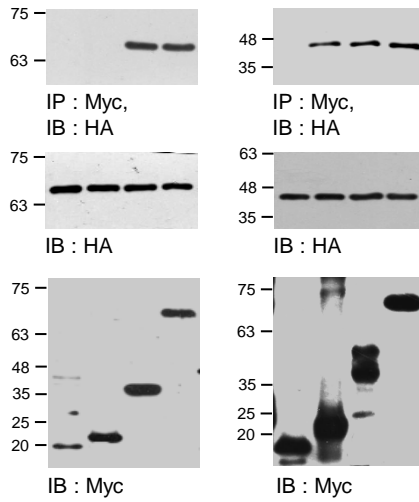

Supplement: Supplementary file 10 [file emmm0007-0577-sd10.pdf]

F

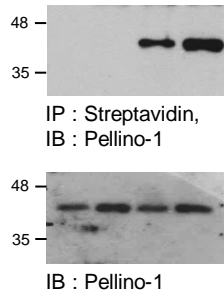

H

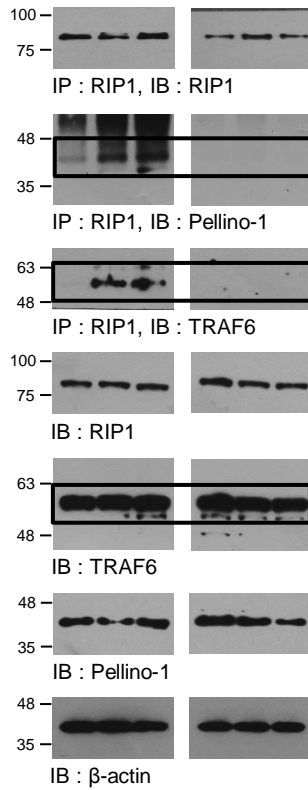

G

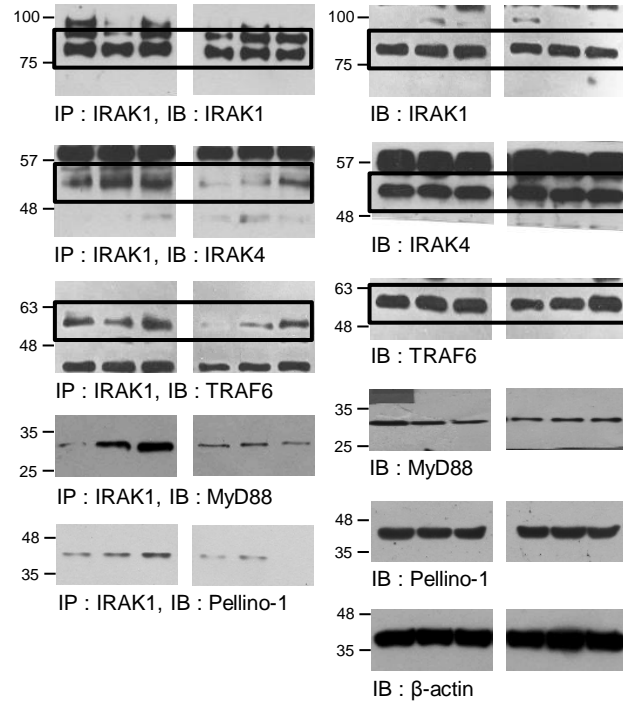

I

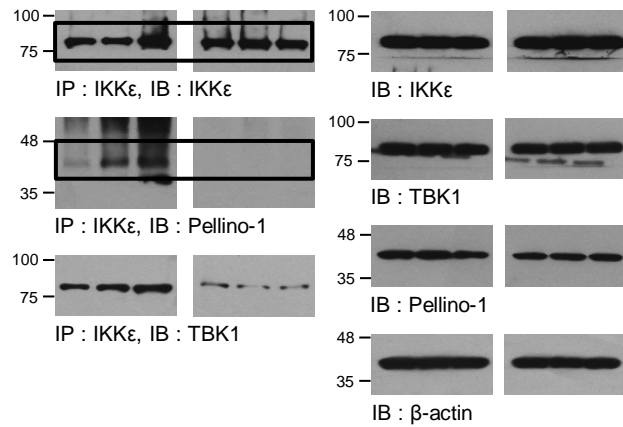

Supplement: Supplementary file 11 [file emmm0007-0577-sd11.pdf]

J

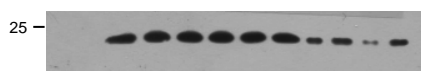

IB : IL-6

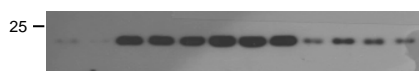

IB : IL-6

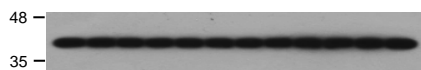IB :  $\beta$ -actin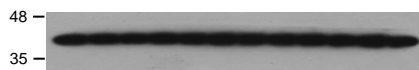IB :  $\beta$ -actin

Supplement: Supplementary file 12 [file emmm0007-0577-sd12.pdf]

D

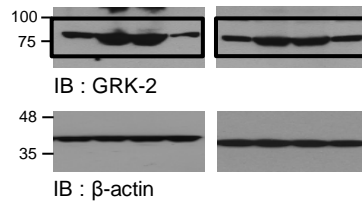

F

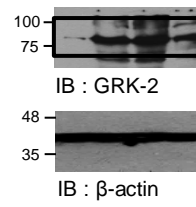

Supplement: Supplementary file 13 [file emmm0007-0577-sd13.pdf]
