# Supplementary material for: Inhibition of lethal inflammatory responses through the targeting of membrane-associated Toll-like receptor 4 signaling complexes with a Smad6-derived peptide
Source: EMBO Mol Med. 2015 Mar 12;7(5):577–92. doi: 10.15252/emmm.201404653 (PMC4492818; doi:10.15252/emmm.201404653)
Supplement: Supplementary file 8 [file emmm0007-0577-sd8.pdf]

EMBO
Molecular Medicine

Inhibition of lethal inflammatory responses through the targeting of membrane-associated Toll-like receptor 4 signaling complexes with a Smad6-derived peptide

Youn Sook Lee, Jin Seok Park, Su Myung Jung, Sang-Doo Kim, Jun Hwan Kim, Jae Young Lee1, Kyeong Cheon Jung, Mizuko Mamura, Sangho Lee, Seong-Jin Kim, Yoe-Sik Bae, Seok Hee Park

Corresponding author: Seok Hee Park, Sungkyunkwan University

Review timeline:

Submission date:	16 September 2014
Editorial Decision:	26 September 2014
Revision received:	12 January 2015
Editorial Decision:	26 January 2015
Revision received:	15 February 2015
Accepted:	19 February 2015

Transaction Report:

(Note: With the exception of the correction of typographical or spelling errors that could be a source of ambiguity, letters and reports are not edited. The original formatting of letters and referee reports may not be reflected in this compilation.)

Editor: Céline Carret

1st Editorial Decision 26 September 2014

Thank you for the submission of your manuscript to EMBO Molecular Medicine. We have now heard back from the two referees whom we asked to evaluate your manuscript. Although the referees find the study to be of potential interest, they also raise a number of concerns that must be addressed in the next version of your manuscript.

As you will see from the comments below, both referees find the study interesting but do have suggestions and recommendations to further improve conclusiveness and clarity as well as increase the potential clinical implications, which is particularly important for our scope.

I will not get into experimental details as the reports are clear and self-explanatory. However, we would strongly encourage you to address all issues raised as recommended, and experimentally as needed.

Given these evaluations, I would like to give you the opportunity to revise your manuscript, with the understanding that the referees' concerns must be fully addressed and that acceptance of the manuscript would entail a second round of review. Please note that it is EMBO Molecular Medicine policy to allow only a single round of revision and that, as acceptance or rejection of the manuscript will depend on another round of review, your responses should be as complete as possible.

EMBO Molecular Medicine has a "scooping protection" policy, whereby similar findings that are published by others during review or revision are not a criterion for rejection. Should you decide to

submit a revised version, I do ask that you get in touch after three months if you have not completed it, to update us on the status.

Please also contact us as soon as possible if similar work is published elsewhere. If other work is published we may not be able to extend the revision period beyond three months.

I look forward to receiving your revised manuscript.

***** Reviewer's comments *****

Referee #1 (Remarks):

The study of Lee et al. is a valuable study that contributes to our understanding of sepsis and may lead to new therapeutic interventions.
Major points
The study uses a quite severe form of CLP, in which mice die within 3 days. The effects of Smaducin should also be validated in less severe conditions. It is also mandatory, in order to make the link to the clinical situation, to test the effect of Smaducin in CLP mouse models which also use antibiotics.
In general, the findings are sound, but the sizes of the groups of mice are rather small. All studies with mouse groups smaller than n=5 must be repeated, even those studies where already statistical significances are reached.
The whole identification of the critical part of Smad-6 is spread over Supplemental figures and Fig 1 and it would make more sense to collect all these data in 1 figure, namely Fig 1. This is better, more logical, more complete and more comfortable for the reader.
In the CLP experiments, mice are treated with a control peptide or with Smaducin-6, but the control CLP mice, I presume, are not treated with PBS or solvent. If this is the case, then this is misleading. Any fluid injection during CLP is a form or resuscitation. It does not suffice to use the control peptide.
To my opinion, taking only one control peptide (as scrambled version of Smaducin-6) is a big risk. The control was not properly validated in binding studies.
In many experiments (e.g. Fig. 4 and Fig.5), there is only one time point measured, lacking dynamics of features). This limits the value of the study.
Page 7: Immunoprecipitation assays showed...: one cannot conclude this. Can only be concluded if Smad6 transfection would lead, in a dose-responsive way, to Pell-Ripk1 or Pell-IKKe interaction loss.
Page 11: The authors should show that Smaducin-6 really leads to reduced Ripk1 and/or IRAK-1 kinase activity.
The tissue distribution studies of Smaducin-6 would only be interesting if the are done with s.c. and i.v. injected material so that pharmacokinetics are obtained, which may explain the lack of effect of the drugs when given by these two routes in mice.
Has it been tested whether Smaducin-6 has direct antibiotic effects?
Fig 6A and 6B are really not useful. There should be found a way to quantify the data and put them in graphs.
When talking of IFNb and its role in CLP, one should refer to Dejager et al, J. Infectious Disease.
The TRAIL data are nice, but should be elaborated a bit more, namely some more readouts that make that link harder.
Smaller issues
In Fig 2A: There is a control missing, namely LPS without anything added.
In Fig. 1C: a control is lacking: only Smaducin-6 without LPS
Fig 3C is almost impossible to see the message without help (arrows).
Fig.3: survival curves. What are these p-values referring to? Can't be found in the legends. I would suggest to calculate p-values of the groups towards the CLP control (which should be CLP + PBS or vehicle) and not towards sham.
Quality of the FACS plots is below the acceptable.
Page 14, 4th line: These results...: this is over-interpretation. You can say that the CLP protective effect is reflected in reduced cytokines, not necessarily due to reduced cytokines.
Fig. 5E: A statistical analysis is lacking
Fig. S3C: TGFb stimulation should be added

Fig/ S7: Label on the figure is wrong: should be LPS instead of CLP. Legend is also wrong: one cannot give four times 100 microgram/kg, adding up to 16 mg/kg
Fig S11: Pal-Scam has some effect. Please explain.

Referee #2 (Remarks):

This paper reports that a lipid modified peptide derived from Smad6 inhibits TLR4 signaling and protects mice from systemic sepsis. The results obtained are interesting and the data shown overall convincing. However, before this paper is considered for publication, the following points should be addressed.

1. The Introduction and the "The paper explained" focus a lot on TGFbeta. Morever, the statement in the Introduction that the anti-inflammatory effects of TGFbeta is specific to the TLR4 pathway seems as an oversimplification. Smad6, which is used as a tool by the authors to inhibit inflammation, inhibits preferentially BMP signaling since it interacts with BMP type I receptors, but not with TGFbeta type I receptors; there is also an inhibitory effect on TGFbeta signaling through the ability of Smad6 to intract with Smad4, which is verified by the authors in this paper. Since the link to TGFbeta is rather weak in this study, the authors should balance the texts of the Introduction and the "The paper explained" sections.

2. The Introduction and Discussion sections are long, and could be shortened without loss of essential information. Moreover, the language of the paper could also be improved.

3. Fig. 1B: This figure is confusing. There is a band at about 15 kDa representing the protein expressed by the Smad6(422-441) vector. Most of the mass of this protein must come from the 6xMyc tag, since the contribution from the Smad6(421-441) peptide would only be a little bit more than 2 kDa. Why is there then no peptide in the pcDNA-6xMyc control?

4. Fig. 1C: An Ig control for the immunoprecipitation is missing in this experiment.

5. In Fig. 1, the effects of the Smad6(421-441) peptide on the SBE-Luc and NF-kB-Luc reporters are shown. Since Smad6 is particularly efficient in inhibiting BMP signaling, it would make sense to include also a BMP reporter construct.

6. The lipid modified Smaducin-6 peptide shows nice inhibitory effects in the in vivo model. Attempts should be made to identify the cell type in which the inhibitory effect occurs, which leads to suppressed inflammation. At a minimum, this should be deiscussed in the Discussion section. Moreover, how is it possible for the lipid modified Smad6-derived peptide to reach its target cell when it after injection is exposed to such vast areas of lymphatic and/or blood endothelial cells?

1st Revision - authors' response 12 January 2015

Referee #1 (Remarks):

Major points

Q1. *The study uses a quite severe form of CLP, in which mice die within 3 days. The effects of Smaducin should also be validated in less severe conditions.*

Answer: We performed the experiments with ten mice (n=10) to assess the therapeutic effects of Smaducin-6 peptide in less severe condition. The new results are included in Supplementary Fig S9C and are described in the Results in the revised manuscript (line 18-19 , page 12).

Q2. *It is also mandatory, in order to make the link to the clinical situation, to test the effect of Smaducin in CLP mouse models which also use antibiotics.*

Answer: As suggested, we examined the synergistic effects of Smaducin-6 and antibiotics after comparing the therapeutic effects of Smaducin-6 and antibiotics. We intraperitoneally injected both gentamycin (8 mg/kg) and cephalosporin (8 mg/kg) into each group twice at 2 h and 14 h post-CLP. When CLP mice, treated with antibiotics alone, showed a 40 % survival rate, treatment of a total of 8 mg/kg Smaducin-6 together with antibiotics increased the survival rate of CLP-induced mice until day 10. In contrast, high-dose Smaducin-6 (12 mg/kg and 16 mg/kg) cancelled out their therapeutic effects. These results indicated that lower concentration of Smaducin-6 is more effective at treating CLP-induced sepsis when treated together with antibiotics.

Although we do not exactly understand this phenomenon, the reason that high concentration of Smaducin-6 together with antibiotics does not show a protective effect at late time points compared to a high concentration of Smaducin-6 alone may be due to the killing effects of cephalosporin which releases high levels of endotoxin and other components from the bacteria (Kirikae et al., Microbiol Immunol. 1997. 41:285-294). When exposed to higher amounts of endotoxins by the treatment with cephalosporin, it might be possible that profound inhibition of Pellino-1-mediated cascade by high-dose Smaducin-6 up regulates other Pellino-1-independent TLR4 inflammatory signalling cascades, whereas partial inhibition of Pellino-1-mediated cascade by low-dose Smaducin-6 synergistically works with bactericidal antibiotics. These findings suggest that elaborated optimization of Smaducin-6 concentration is required for combined treatment with antibiotics in clinical situations, although a higher concentration of Smaducin-6 alone is effective in CLP-induced sepsis mice model. The new results are included in Fig 4C and 4D and their clinical implications are described in the Results and Discussion in the revised manuscript (line 3-16, page 13; line 8-22, page 22).

Q3. *In general, the findings are sound, but the sizes of the groups of mice are rather small. All studies with mouse groups smaller than n=5 must be repeated, even those studies where already statistical significances are reached.*

Answer: All the animal experiments used more than n=5 mice, except for the FACS analysis for Fig 5E and Supplementary Fig S11 in the original manuscript. The FACS analysis to detect CXCR2-expressing neutrophils were repeated with five mice (n=5) per group and statistically analysed. The new results were presented in Fig. 6E in the revised manuscript and the number of mice were described in the figure legend of Fig 6E. The animal experiments regarding Q1 and Q2 were also performed with ten mice and statistically analysed and all typos regarding the numbers of mice were corrected in the revised manuscript.

Q4. *The whole identification of the critical part of Smad-6 is spread over Supplemental figures and Fig 1 and it would make more sense to collect all these data in 1 figure, namely Fig 1. This is better, more logical, more complete and more comfortable for the reader.*

Answer: As suggested, the results in Supplementary Fig S2 and Fig S3 in the original manuscript were collected as a new Fig 1 in the revised manuscript. Fig 1 in the original manuscript was reordered as Fig 2 in the revised manuscript.

Q5. *In the CLP experiments, mice are treated with a control peptide or with Smaducin-6, but the control CLP mice, I presume, are not treated with PBS or solvent. If this is the case, then this is*

misleading. Any fluid injection during CLP is a form or resuscitation. It does not suffice to use the control peptide.

Answer: All the control CLP mice were actually treated with PBS in the original manuscript. In the revised manuscript, we described the injection of PBS into the control CLP mice as CLP + PBS in the new figures and described the injection of PBS into the control CLP mice in the Results.

Q6. *To my opinion, taking only one control peptide (as scrambled version of Smaducin-6) is a big risk. The control was not properly validated in binding studies.*

Answer: We agree with the reviewer's comment. We have synthesized two additional scrambled peptides with different amino acid sequences, named Pal-Scram #2 and Pal-Scram #3, in the revised manuscript. The control Pal-Scram peptide in the original manuscript was renamed Pal-Scram #1. We next confirmed the direct interactions of these control peptides with endogenous Pellino-1 by a biotin-streptavidin pull-down assay. Biotin-labelled Pal-Scram peptides did not interact with endogenous Pellino-1 whereas Smaducin-6 did. These results are added in Supplementary Fig S9B and described in the Results in the revised manuscript. Furthermore, to exclude the possibility that non-specific effects of the peptide might affect the survival rate of CLP mice, we investigated the survival rates of CLP-induced mice which were injected with one of three scrambled peptides (Pal-Scram #1, Pal-Scram #2 and Pal-Scram #3). CLP mice injected with different scrambled peptides died within 3 days, similar to the original results with mice injected with the Pal-Scram #1 peptide in the original manuscript. These results indicated that these additional Pal-Scram peptides are valid as negative controls and supported the therapeutic effect of Smaducin-6 on CLP-induced sepsis mice. These survival data are included in Supplementary Fig S7A and described in the Results in the revised manuscript. Amino acid sequences of the scrambled peptides are included in Supplementary Table S1.

Q7. *In many experiments (e.g. Fig. 4 and Fig.5), there is only one time point measured, lacking dynamics of features). This limits the value of the study.*

Answer: We examined the changes of pro-inflammatory cytokines, IL-6, TNF-alpha, IFN-gamma, and IL-1beta, of which systemic up regulation is an important characteristic, at different time points. In both blood and peritoneal fluid, ELISA assays indicated that Smaducin-6, subcutaneously injected after CLP, significantly inhibited the expression of pro-inflammatory cytokines past 12 h after CLP. For these experiments, we used five mice (n=5) per group. These results are included in Supplementary Fig S12 and described in the Results in the revised manuscript (line 6-9, page 14).

Q8. *Page 7: Immunoprecipitation assays showed...: one cannot conclude this. Can only be concluded if Smad6 transfection would lead, in a dose-responsive way, to Pell-Ripk1 or Pell-IKKe interaction loss.*

Answer: To confirm whether Smad6 disrupts Rip1-mediated or IKKε-mediated signalling complexes through direct binding to Pellino-1, we performed immunoprecipitation assays while increasing expression of ectopic Flag-Smad6. As Smad6 increased, Smad6 binding with endogenous Pellino-1 was augmented and thus the Pellino-1-RIP1 complex and Pellino-1-IKKε complex, induced by LPS treatment, were disrupted in a dose-dependent manner. These results are added in Supplementary Fig. 1C and described in the Results in the revised manuscript.

Q9. *Page 11: The authors should show that Smaducin-6 really leads to reduced Ripk1 and/or IRAK-1 kinase activity.*

Answer: Although RIP1 kinase has been extensively studied in the activation of cell deaths caused by TNF-alpha including apoptosis and necroptosis, it is also known as an important regulator that controls inflammatory signalling following the activation of TLR3 or TLR4 (Ofengeim and Yuan, Nat. Rev. Mol. Cell Biol. 2013. 14:727-736). However, it is still controversial whether RIP1 kinase activity is required for NF-κB activation. Several reports indicated that a kinase-dead RIP1 mutant and the pharmacological inhibition of RIP1 kinase activity by necostain-1 have no effect on the activation of NF-κB (Lee et al., J. Biol. Chem. 2004. 279:33185-33191; Degterev et al., Nat. Chem. Biol. 2008. 4:313-321). In contrast, a certain report showed that the production of TNF is dependent on RIP1 kinase activity (Christofferson et al., Cell Death Dis. 2012. 3:e320). However, the more important finding regarding the activation of RIP1 in the TLR pathway was that Pellino-1 induces the ubiquitination of RIP1, which ubiquitination is critical for activation of the downstream signalling pathway. These previous reports mean that it is likely valuable for us to examine whether binding of the Smaducin-6 peptide to Pellino-1 affects RIP1 ubiquitination. Therefore, we examined whether Smaducin-6 decreases RIP1 ubiquitination through binding to Pellino-1 but not the activity of the RIP1 kinase. HEK293 cells were pre-treated with 100 nM scrambled peptide (Pal-Scram #1) or Smaducin-6 for 2 h, and plasmids encoding HA-tagged ubiquitin, Flag-RIP1, and Flag-Pellino-1 were transitently co-transfected. The scrambled peptide did not affect RIP1 ubiquitination whereas Smaducin-6 significantly decreased RIP1 ubiquitination. These results are caused because Smaducin-6 binding with Pellino-1 disrupts the Pellino-1-RIP1 complex. The new results are included in Supplementary Fig S6 and described in the Results with references in the revised manuscript (line 9-19, page 10).

We next examined the phosphorylation of IRAK1 in the presence of Smaducin-6 by phospho-IRAK1 antibody. As a result, Smaducin-6 disrupted the LPS-induced formation of the IRAK1-mediated signalling complex but did not inhibit IRAK1 phosphorylation. Until now, it has not been clearly addressed whether Pellino-1 regulates the phosphorylation of IRAK1. Although Pellino-1 phosphorylation by IRAK1 was reported to increase polyubiquitination of IRAK1 as well as Pellino-1 *in vitro* (Butler et al., J. Biol. Chem. 2007. 282:29729-29737; Ordureau et al., Biochem. J. 2008. 409:43-52), the mechanisms between the phosphorylation and ubiquitination of Pellino-1 and IRAK1 have not been verified *in vivo*. Thus our results collectively suggest that the major function of Smaducin-6 is to interfere with formation of the Pellino-1-mediated signalling complex through direct binding to Pellino-1 and indicate the presence of an unknown enzyme, independent of Pellino-1, that regulates IRAK1 phosphorylation. These results are included in Supplementary Fig S15 and described in the Discussion with references in the revised manuscript (line 20, page 19 - line 4, page 20).

Q10. *The tissue distribution studies of Smaducin-6 would only be interesting if the are done with s.c. and i.v. injected material so that pharmacokinetics are obtained, which may explain the lack of effect of the drugs when given by these two routes in mice.*

Answer: We repeated the experiments to analyse tissue distribution of the fluorescence-conjugated Smaducin-6 peptide, when it was injected intravenously, together with subcutaneous injection. Subcutaneous injection resulted in much more effective tissue distribution compared with intravenous injection (Supplementary Fig. S8 in the revised manuscript). As the reviewer suggested, we used these results to explain the higher efficacy of subcutaneous injection than that of intravenous injection (line 15, page 11 - line 4, page 12).

Q11. *Has it been tested whether Smaducin-6 has direct antibiotic effects?*

Answer: We examined the direct killing effect of Smaducin-6 against gram negative bacteria, *E. coli*. We did not observe any direct antibiotic effects against *E. coli* DH5α, when we compared it to ampicillin (50 μg/ml) treatment. The results are included in Supplementary Fig S13B and described in the Results in the revised manuscript.

***Q12**. Fig 6A and 6B are really not useful. There should be found a way to quantify the data and put them in graphs.*

Answer: In the TUNEL assay (Fig. 6A) and IHC of Caspase-3 (Fig. 6B), at least five hot spots in a section per experiment were selected, average counts were determined and the data were described in bar graphs. Data were expressed as a mean of positive cells per area and statistically analysed by the *t*-test. The results are included in Fig 7A and 7B in the revised manuscript. Fig 6A and 6B in the original manuscript were moved to Supplementary Fig. S14 in the revised manuscript.

***Q13**. When talking of IFNb and its role in CLP, one should refer to Dejager et al, J. Infectious Disease.*

Answer: We added the reference in the revised manuscript.

***Q14**. The TRAIL data are nice, but should be elaborated a bit more, namely some more readouts that make that link harder.*

Answer: We substantially described the previous finding about how TRAIL, produced by $CD8^+$ regulatory T cells, contributes to immune suppression during sepsis and added the related references in the Results and Discussion in the revised manuscript.

Smaller issues

***Q15**. In Fig 2A: There is a control missing, namely LPS without anything added.*

Answer: We already had the data where RAW264.7 cells were treated with LPS only. We replaced Fig 2A with the revised results (Fig 3A) including only LPS treatment in the revised manuscript.

***Q16**. In Fig. 1C: a control is lacking: only Smaducin-6 without LPS*

Answer: We think that the reviewer is pointing to Fig 2C, and not Fig 1C in the original manuscript.

In Fig. 2C in the original manuscript, Smaducin-6 was pre-treated for 30 min. The effects of Smaducin-6 only treated without LPS were shown at the zero time point of Fig 2C in the original manuscript. Therefore, we could conclude that Smaducin-6 did not affect the expression of IκBα, IKKα, and phospho-IKKα/β, compared to the zero-time point of the Pal-Scram #1 peptide. Fig 2C was re-named Fig 3C in the revised manuscript.

***Q17**. Fig 3C is almost impossible to see the message without help (arrows).*

Answer: We replaced Fig. 3C of the original manuscript with a new Fig 4B with better resolution in the revised manuscript. Also, we demonstrated the following pathological characteristics, pointed out as arrows or circles, in the revised manuscript. Hematoxylin and eosin (H/E) staining in several types of tissue from CLP mice or the scrambled peptide-treated CLP mice revealed severe pulmonary inflammation with alveolar wall thickening, and necrosis of hepatocytes (arrow: increased eosinophilia of cytoplasm and pyknosis) and splenocytes (circle: karyorrhexis), whereas Smaducin-6 treatment significantly reduced these alterations.

© EMBO

Q18. *Fig.3: survival curves. What are these p-values referring to? Can't be found in the legends. I would suggest to calculate p-values of the groups towards the CLP control (which should be CLP + PBS or vehicle) and not towards sham.*

Answer: We corrected the p-values of all survival data in the revised manuscript, re-calculating them toward CLP mice injected with Pal-scrambled peptide or PBS as a vehicle (CLP+Pal-Scram #1 or CLP+PBS). The way we calculated the p-values are described in the figure legends in the revised manuscript.

Q19. *Quality of the FACS plots is below the acceptable.*

Answer: The FACS plots shown in Fig 5C, Fig 6D, and Fig 6E were replaced with FACS plots with better resolution in the revised manuscript. The new FACS plots are re-named Fig 6C, Fig 7D, and Fig 7E in the revised manuscript.

Q20. *Page 14, 4th line: These results...: this is over-interpretation. You can say that the CLP protective effect is reflected in reduced cytokines, not necessarily due to reduced cytokines.*

Answer: We rephrased the indicated sentence into the following: " Reduction in pro-inflammatory cytokine levels in peripheral blood and peritoneal fluid explains the protective effect of Smaducin-6 on sepsis"

Q21. *Fig. 5E: A statistical analysis is lacking*

Answer: The FACS and statistical analysis were performed using neutrophils obtained from each group (n=5 mice/group). The new data with statistical significance are included in Fig 6E in the revised manuscript.

Q22. *Fig. S3C: TGFb stimulation should be added*

Answer: We already had the data regarding the effects of TGF-β1 pre-treatment on the expression of IκBα, IKKα, the phospho-IKKα/β, and we added it to the original Fig S3C. The new results are renamed Fig 1E in the revised manuscript and described in the Results in the revised manuscript.

Q23. *Fig/ S7: Label on the figure is wrong: should be LPS instead of CLP. Legend is also wrong: one cannot give four times 100 microgram/kg, adding up to 16 mg/kg*

Answer: We corrected the typo in the revised manuscript.

Q24. *Fig S11: Pal-Scam has some effect. Please explain.*

Answer: We think the effect is likely to be an artifact of FACS analysis. To exclude these artifacts, the FACS analysis regarding CXCR2-expressing neutrophils were repeated again with five mice (n=5) per group and statistically analysed. The new results were described in bar graphs with p values. The results are included in Fig 6E in the revised manuscript and the number of mice are described in the figure legend. Also, the original Supplementary Fig S11 was deleted from the revised manuscript.

Referee #2 (Remarks):

***Q1**. The Introduction and the "The paper explained" focus a lot on TGFbeta. Moreover, the statement in the Introduction that the anti-inflammatory effect of TGFbeta is specific to the TLR4 pathway seems as an oversimplification. Smad6, which is used as a tool by the authors to inhibit inflammation, inhibits preferentially BMP signalling since it interacts with BMP type I receptors, but not with TGFbeta type I receptors; there is also an inhibitory effect on TGFbeta signalling through the ability of Smad6 to interact with Smad4, which is verified by the authors in this paper. Since the link to TGFbeta is rather weak in this study, the authors should balance the texts of the Introduction and the "The paper explained" sections.*

Answer: During the revision of our manuscript, we found that the minimal region of Smad6 binding to Pellino-1 did not interact with Smad4 whereas the full-length Smad6 still bounds to Smad4. These results demonstrate the reason that the minimal region of Smad6 can inhibit only LPS-induced TLR4 signalling. Therefore, we tried to write a balanced text about Smad6 having an anti-inflammatory role in TLR4 signalling as well as acting as a negative regulator of TGF-beta/BMP signalling in the Introduction and Results in the revised manuscript. The new results that the minimal region of Smad6 does not bind to Smad4 but only Pellino-1 are included in Fig 2G in the revised manuscript. Also, we re-wrote "Abstract" and "The paper explained", as following the reviewer's advice.

***Q2**. The Introduction and Discussion sections are long, and could be shortened without loss of essential information. Moreover, the language of the paper could also be improved.*

Answer: We tried to shorten the Introduction and Discussion and adjusted total word counts, including spaces, to 60,000 characters, according to the policy of EMBO Mol Med. To adjust total word counts, parts of the "Materials and Methods" in the original manuscript are moved to "Supplementary Information" in the revised manuscript. We also did our best to improve the language of the paper with the help of a native English-speaking scientist.

***Q3**. Fig. 1B: This figure is confusing. There is a band at about 15 kDa representing the protein expressed by the Smad6(422-441) vector. Most of the mass of this protein must come from the 6xMyc tag, since the contribution from the Smad6(421-441) peptide would only be a little bit more than 2 kDa. Why is there then no peptide in the pcDNA-6xMyc control?*

Answer: We agree with the reviewer's comment. The size markers were labelled incorrectly. We performed the immunoprecipitation and immunoblot analysis again. The empty vector, pCS3MTBXA-6xMyc, was transfected with HA-Pellino-1 as a control. This empty vector consists of six Myc epitopes, flanking sequences, multicloning sites, and stop codon. There are 147 additional flanking amino acids, including six Myc epitopes, in this empty vector which are observed at a size of 16 kD-20 kD, depending on the percentage of SDS-PAGE gel. The 6xMyc-Smad6(422-441) plasmid includes 20 additional amino acids, compared to the empty vector. The expression of 6xMyc-Smad6(422-441) was observed as a size of about 18-22 kD. The new figures are included in Fig 2B in the revised manuscript.

***Q4**. Fig. 1C: An Ig control for the immunoprecipitation is missing in this experiment.*

Answer: The experiment of Fig 1C in the original manuscript was repeated again. The IgG control antibody was added to this experiment as a negative control. The new results are included in Fig 2C in the revised manuscript.

© EMBO

***Q5**. In Fig. 1, the effects of the Smad6(421-441) peptide on the SBE-Luc and NF-kB-Luc reporters are shown. Since Smad6 is particularly efficient in inhibiting BMP signalling, it would make sense to include also a BMP reporter construct.*

Answer: As suggested, we tested the activity of the BRE (BMP response element)-Luc reporter in the presence of full-length Smad6 or Smad6(422-441) expression to examine whether the Smad6(422-441) expression affects the BMP signalling. We treated RAW264.7 cells with several BMP ligands, including BMP2, BMP4 and BMP6, after the BRE-Luc reporter plasmid was transfected with the full-length Smad6 or Smad6(422-441) plasmid. Smad6(422-441) expression did not inhibit BMP signalling whereas Smad6 significantly inhibited BMP signalling. Furthermore, co-immunoprecipitation assays indicated that the Smad6 MH2 domain, including Smad6(422-441) region, specifically binds to Smad4, as reported by Moren et al (JBC, 2005. 280: 22115-22123). However, the minimal region of Smad6(422-441) did not bind to Smad4, and only bound to Pellino-1. These results suggest that Smad6(422-441) uniquely inhibits TLR4 signalling through binding to Pellino-1. Therefore, the failure of Smad6(422-441) binding to Smad4 demonstrates the reason that this region did not affect TGF-beta/BMP signalling. The new results are included in Fig 2F, Fig 2G and Supplementary Fig S3, and described in the Results of the revised manuscript.

***Q6**. The lipid modified Smaducin-6 peptide shows nice inhibitory effects in the in vivo model. Attempts should be made to identify the cell type in which the inhibitory effect occurs, which leads to suppressed inflammation. At a minimum, this should be discussed in the Discussion section. Moreover, how is it possible for the lipid modified Smad6-derived peptide to reach its target cell when it after injection is exposed to such vast areas of lymphatic and/or blood endothelial cells?*

Answer: The reviewer makes a valid point. The Smaducin-6 peptide was designed based on pepducins, which are cell-penetrating lipidated peptides targeting the intracellular loops of G protein-coupled receptors (Covic et al., Nat Med. 2002. 8:1161-1165; Tressel et al, Methods Mol Biol. 2011. 683: 259-275). Although we have extensively searched all published papers regarding the reviewer's comments, it appears unknown which cell types in animal tissues are influenced by i.v. or s.c. injected pepducin peptides in animal models. The only finding available are the results of the tissue distribution of pepducin peptides, which are mostly distributed to highly vascularized tissues such as kidneys, lungs, spleen, and liver (Tressel et al, Methods Mol Biol. 2011. 683: 259-275). Smaducin-6 also shows similar distribution to pepducin.

Considering the bio distribution of pepducin and our present results of Smaducin-6 tissue distribution, it is possible that s.c injected Smaducin-6 may be delivered through lymphatic vessels and its bio distribution to highly vascularized tissues may be caused by its capability to easily pass through vascular tissue. During this delivery process, Smaducin-6 may target innate immune cells infiltrating injured tissues and may also target immune cells within lymphatic or blood vessels as well as endothelial cells. However, this scenario is speculative. We believe that identifying the target cells of Smaducin-6 in an animal model and the method that it reaches the target cells is beyond the scope of our present manuscript. We included the speculations above in the Discussion of the revised manuscript (line 19, page 21 - line 7, page 22).

2nd Editorial Decision 26 January 2015

Thank you for the submission of your revised manuscript to EMBO Molecular Medicine. We have now received the enclosed reports from the referees that were asked to re-assess it. As you will see the reviewers are now globally supportive and I am pleased to inform you that we will be able to accept your manuscript pending some editorial amendments.

Please submit your revised manuscript within two weeks. I look forward to seeing a revised form of your manuscript as soon as possible.

***** Reviewer's comments *****

Referee #1 (Remarks):

The authors have convincingly replied to my concerns and addressed the issues. Only one more remark: the authors must change the terminology in their abstract and paper: LPS (or endotoxin) induces endototxemia, NOT septic shock.

Referee #2 (Remarks):

The authors¥revision is appropriate, and this paper is now suitable for publication.

© EMBO
